# E-Cadherin Acts as a Regulator of Transcripts Associated with a Wide Range of Cellular Processes in Mouse Embryonic Stem Cells

**DOI:** 10.1371/journal.pone.0021463

**Published:** 2011-07-14

**Authors:** Francesca Soncin, Lisa Mohamet, Sarah Ritson, Kate Hawkins, Nicoletta Bobola, Leo Zeef, Catherine L. R. Merry, Christopher M. Ward

**Affiliations:** 1 Core Technology Facility, Faculty of Medical and Human Sciences, The University of Manchester, Manchester, United Kingdom; 2 Materials Science Centre, The University of Manchester, Manchester, United Kingdom; 3 Microarray Facility, Faculty of Life Sciences, Michael Smith Building, The University of Manchester, Manchester, United Kingdom; University of Southern California, United States of America

## Abstract

**Background:**

We have recently shown that expression of the cell adhesion molecule E-cadherin is required for LIF-dependent pluripotency of mouse embryonic stem (ES) cells.

**Methodology:**

In this study, we have assessed global transcript expression in E-cadherin null (Ecad-/-) ES cells cultured in either the presence or absence of LIF and compared these to the parental cell line wtD3.

**Results:**

We show that LIF has little effect on the transcript profile of Ecad-/- ES cells, with statistically significant transcript alterations observed only for Sp8 and Stat3. Comparison of Ecad-/- and wtD3 ES cells cultured in LIF demonstrated significant alterations in the transcript profile, with effects not only confined to cell adhesion and motility but also affecting, for example, primary metabolic processes, catabolism and genes associated with apoptosis. Ecad-/- ES cells share similar, although not identical, gene expression profiles to epiblast-derived pluripotent stem cells, suggesting that E-cadherin expression may inhibit inner cell mass to epiblast transition. We further show that Ecad-/- ES cells maintain a functional β-catenin pool that is able to induce β-catenin/TCF-mediated transactivation but, contrary to previous findings, do not display endogenous β-catenin/TCF-mediated transactivation. We conclude that loss of E-cadherin in mouse ES cells leads to significant transcript alterations independently of β-catenin/TCF transactivation.

## Introduction

E-cadherin is a member of the classical cadherin family and is expressed on most epithelial cells, including ES cells [1], [2]. The extracellular domain of E-cadherin interacts in a homophilic calcium-dependent manner with E-cadherin molecules on neighbouring cells, thereby facilitating cell-cell contact [1], [3]. E-cadherin is essential for embryogenesis since E-cadherin null embryos fail to develop beyond the blastocyst stage [2], reflecting loss of epithelial integrity in both the trophectoderm and inner cell mass [2], [4]. Loss of cell surface E-cadherin is a defining characteristic of epithelial-mesenchymal transition (EMT), which is required for ingression of epiblast cells within the primitive streak during early embryonic development [1], [5] and is associated with tumour cell metastasis [6], [7]. The cytoplasmic region of E-cadherin binds to β-catenin, allowing interaction with the actin cytoskeleton via the intermediate protein α-catenin and, most likely, Epithelial Protein Lost In Neoplasm (EPLIN) [8]. In addition, p120-catenin binds to the juxta-membrane region of the E-cadherin cytoplasmic domain and contributes to stabilisation of the cadherin-catenin complex by preventing clathrin-mediated endocytosis [9]. Besides its structural role at the cell membrane, β-catenin can also function as a transcriptional regulator in response to Wnt signals [10]. Under normal conditions, β-catenin protein turnover is regulated by a specific complex formed by Glycogen Synthase Kinase 3β (GSK3β), APC protein and Axin, leading to proteosomal degradation. Upon Wnt activation, triggered by numerous Wnt proteins interacting with Frizzled receptors, β-catenin degradation is inhibited and the protein is translocated into the nucleus where it interacts with the T-Cell Transcription Factor/Lymphoid Enhancer-binding Factor (TCF/LEF) complex and regulates expression of Wnt target genes.

During development E-cadherin is regulated spatio-temporally, allowing cellular migration and morphogenesis [1]. Abnormal canonical Wnt activity has been associated with malignant progression of epithelial cancers, in particular gastric tumours, and, in some cases, this transformation has been associated with loss of cell-cell contact via down-regulation of E-cadherin [11]. Forced expression of E-cadherin has been shown to sequester β-catenin protein and negatively interfere with its transcriptional function whilst E-cadherin down-regulation in epithelial cells has been implicated in higher β-catenin-mediated transactivation [12]. Canonical Wnt signalling has also been associated with maintenance of pluripotency in both mouse and human ES cells [13], [14]. Sato et al. [13] showed that culture of mouse ES cells with the GSK3 inhibitor BIO resulted in maintenance of pluripotency in these cells in the absence of Leukaemia Inhibitory Factor (LIF). In addition, Miyabayashi and colleagues have described a method for preventing spontaneous differentiation of ES cells using the small molecule IQ-1, which increases β-catenin/CBP-mediated transcriptional activation [15]. Recently, mouse ES cells have been derived from blastocysts using a chemically defined medium containing Fibroblast Growth Factor 2 (FGF2), Activin A and BIO [16]. However, β-catenin-mediated signalling is not necessary for the maintenance of ES cell pluripotency factors Nanog, Sox2 and Oct3/4 as β-catenin-/- mouse ES cells have been isolated and cultured successfully *in vitro* exhibiting dependency on the Activin/Nodal and FGF cascades rather than LIF/BMP (Bone Morphogenic Factor) [17]. Therefore, whilst β-catenin appears dispensable for the maintenance of pluripotency and self-renewal of ES cells, it is still unclear whether β-catenin-mediated transactivation plays a functional role in E-cadherin-/- ES cells.

As well as acting as an inhibitor of signalling by sequestering catenins, E-cadherin also plays a role in repressing ligand activation of receptor tyrosine kinases (RTKs) such as EGFR, Neu/ErbB2, insulin-like growth factor receptor IGF1R and c-Met [3], [18]. Interestingly, some of these kinases have been associated with induction of EMT in epithelial cells following activation of E-box binding repressors of E-cadherin, such as Snail [19]. A further binding partner of E-cadherin is PIPKIγ, which may play a role in maintaining integrity of the cadherin/catenin complex since ablation of PIPKIγ in human epithelial cells results in loss of plasma membrane localisation of E-cadherin [3]. Moreover, tyrosine phosphorylation of PI(3)K by c-Src leads to the recruitment and interaction of the former with the E-cadherin/β-catenin complex resulting in activation of Akt [20], [21]. Recruitment of PI3K by the E-cadherin/catenins complex is also fundamental for the calcium-induced differentiation of human keratinocytes [22]. Therefore, E-cadherin plays a significant role in both positively- and negatively- regulating exogenous signals to maintain epithelial cell integrity.

We have previously demonstrated that differentiation of ES cells is associated with an EMT-like event, including an E-cadherin to N-cadherin switch at the cell surface and the upregulation of various metalloproteinases (MMPs) [6], [7]. In addition, we have recently shown that abrogation of E-cadherin in mouse ES cells results in modification of the cell response to exogenous factors [17]. For example, E-cadherin-depleted cells exhibit LIF-independent self-renewal and differentiation was induced upon Activin/Nodal signalling pathway inhibition. In this study, we further elucidate the role of LIF in Ecad-/- ES cells by comparing the gene transcription profiles of the cells grown in the presence and absence of LIF. We have also investigated the impact of E-cadherin depletion in ES cells by analysing global gene transcript expression in wtD3 and Ecad-/- ES cells cultured under standard LIF-conditions. In addition, we have investigated β-catenin/TCF transactivation in Ecad-/- ES cells to determine whether this complex is intrinsically active within these cells. In summary, we show that absence of E-cadherin in ES cells results in significant alterations in transcript expression in a wide range of cellular processes compared to wtES cells. In addition, we demonstrate that LIF exerts a limited effect on the transcriptional profile of Ecad-/- ES cells and that these cells do not display endogenous β-catenin/TCF-mediated transactivation.

## Materials and Methods

### Cell culture

Mouse D3 (129/Sv+c/+p), E-cadherin-/- (derived from D3 ES cells; a kind gift from Professor Rolf Kemler, Germany) and MESC20 (129/OLA) ES cells were cultured on gelatinised plates in knockout D-MEM medium containing 10% (v/v) Foetal Calf Serum (FCS), 2mM L-glutamine, 1% (v/v) non-essential amino acids and 0.1% (v/v) 2-mercaptoethanol (all Invitrogen, Paisley, UK) and 1000U/ml LIF (ESGRO, Millipore, Watford, UK) as previously described [17]. Ecad-/- ES cells were also cultured for 12 days in the absence of LIF under a normal passaging regimen prior to experimental analysis. All ES cells presented in this study exhibited normal karyotypes.

### Wnt activation reporter system

To analyse the level of canonical Wnt activation in wtD3 and Ecad-/- ES cells, 2 µg of the Wnt reporter plasmid TOPflash or the relative negative control FOPflash (Millipore, Watford, UK) were transfected into ES cells using the mouse ES cell Nucleofector® system (Amaxa Biosystems, Wokingham, UK) as previously described [17]. 0.5 µg of a *Renilla* plasmid (Promega, Southampton, UK) was transfected together with the reporter plasmids and used for normalisation in the Dual-Luciferase Reporter assay. After transfection, cells were cultured in standard medium or with addition of the GSK3 inhibitor BIO at a final concentration of 2 µM and 5 µM for wtD3 ES cells and 1 µM and 2 µM for Ecad-/- ES cells (or equivalent volume of DMSO as control). A Dual-Luciferase Reporter assay (Promega, Southampton, UK) was performed 24h after transfection according to the manufacturer's instructions. The assay was performed on the automatic Berthold Detection System and the Luciferase/*Renilla* luminescence ratio was calculated for each well. Results show mean ± SD of one single experiment; at least three independent experiments were performed.

### Quantitative assay for β-catenin protein

1×10^5^ cells/well were seeded into a gelatinised 96-well plate and cultured for 24h as described above and fixed overnight at 4°C with 4% (w/v) paraformaldehyde (PFA, Sigma, Dorset, UK) in PBS. Imagen Biotech (Manchester, UK) performed a β-catenin immunofluorescence quantification assay according to the following optimised protocol. Cells were stained with the primary anti-β-catenin antibody (1∶100, Abcam ab6302, Cambridge, UK), diluted in 500 µg/ml digitonin/PBS at room temperature for 1h. Secondary antibody goat anti-rabbit Alexa488-conjugated diluted 1∶500 in PBS with addition of 25 µg/ml Hoescht solution (Invitrogen, Paisley, UK) was incubated for 30min at room temperature. After further fixation in 1% formaldehyde for 15min, cells were kept in PBS and visualised using a Cellomics Arrayscan. A specific Arrayscan algorithm was used to quantify β-catenin in the various cellular compartments (nucleus, cytoplasm and cell membrane). To distinguish between transcriptionally active and inactive β-catenin protein pools, the protein in the cytoplasmic and the membrane compartments are presented together (cytoplasm+membrane).

### Microarray analysis

wtD3 and Ecad-/- ES cells were grown in standard medium +LIF and Ecad-/- ES cells were also cultured in the absence of LIF for 12 days under a normal passaging regimen. RNA was extracted as previously described [17] and microarray analysis performed by the Microarray Facility of Life Sciences (The University of Manchester, UK) using Affymetrix genome Mouse 430 v.2 chips. Technical quality control was performed with dChip (V2005) (www.dchip.org) using default settings. Background correction, quantile normalization, and gene expression analysis were performed using GCRMA in Bioconductor [23]. A dendrogram of array relationships was performed by hierarchical clustering (Euclidean distance) with Partek Genomics Solution (version 6.5, Copyright 2005, Partek Inc., St. Charles, MO, USA). After normalisation, the fold-change (FC) expression was calculated between Ecad-/- grown in LIF and wtD3 ES cells and between Ecad-/- ES cells cultured in the presence and absence of LIF. Differential expression analysis was performed using Limma using the functions lmFit and eBayes [24] and false discovery rate (fdr) errors controlled using the method of QVALUE [25]. A q value (q<0.050) and the fold-change threshold FC≥2.50 was chosen to identify the statistically significant transcript alterations. The website DAVID was used to analyse the enrichment in the Gene Onthology (GO) terms and KEGG Pathway terms among the statistically significant genes between Ecad-/- and wtD3 ES cells [26], [27]. The software GO-Elite was used to analyse the Wikipathways represented in the statistically significant gene list (z score ≥2.0) (www.genmapp.org/go_elite). The microarray data is published in ArrayExpress, accession number E-MEXP-2836, and all data is MIAME compliant. Agilent array platform data for EpiSCs [28] was obtained from GEO (GSE7902-GPL4134). The raw data was quantile normalised and differential expression assessed between ES and post-ES groups by t-test and fdr by QVALUE using Partek Genomics Solution (version 6.5, Copyright 2005, Partek Inc.). Data was merged with our dataset using the gene symbol annotation using the Galaxy platform [29]. Both array platforms have multiple measurements for some genes. Where this was the case, the measurement showing the most highly significant p-value in differential expression was used in merging the data.

### RT-PCR

Total RNA was extracted from cells using Trizol (Sigma) according to the manufacturer's instructions, treated with DNase (Promega) and phenol/chloroform purified. Synthesis of cDNA was performed as described previously [30]. RT-PCR was performed using 1 µl of the cDNA solution and amplified for 30 cycles at optimal annealing temperature. Samples were separated on 2% (w/v) agarose gels containing 400 ng/ml ethidium bromide and visualised using a UV transilluminator. Primer sequences are shown in Table S1.

### qPCR

Quantitative PCR was used to confirm the microarray results. Expression of genes related to EpiS cells was analysed using MRP L19 as a housekeeping gene. The qPCR primers were designed using the Universal Probe Library software on the Roche Applied Science website. cDNA was made from 2 µg of RNA, as previously described [17], and diluted 1∶100 with nuclease-free water for qPCR amplification. Each cDNA sample/primer set combination was run in triplicate in 96-well MicroAmp Optical reaction plates (Applied Biosystems, Warrington, UK). Mouse genomic DNA (gDNA, Bioline, London, UK) was used as positive control whilst water and the –RT reaction were used to check for false-positives and contaminants on each sample using MRP L19. Primer sequences are shown in Table S2. Three independent RNA samples were analysed for both wtD3 and Ecad-/- ES cells. A typical qPCR reaction contained: 5 µl of diluted cDNA, 0.075 µl of each forward and reverse primer at 100 µM concentration stock, 12.5 µl Power SYBR Green PCR Master Mix (AB Applied Biosystems, Warrington, UK) and 7.35 µl nuclease-free water. The qPCR reaction was performed on a 7300 Real Time PCR System (AB Applied Biosystems, Warrington, UK) and comprised 40 cycles of PCR at 60°C annealing temperature and at the end the dissociation curve stage was performed. The Ct values were exported into a Microsoft Excel Spreadsheet and analysed according to the ΔCt system. The –ΔΔCt (Ecad-/-vs wtD3) values were plotted to show the genes that are up or downregulated in Ecad-/- ES cells compared to wtD3 ES cells.

## Results

### LIF supplementation does not significantly alter the transcript profile of Ecad-/- ES cells

**Figure 1:**
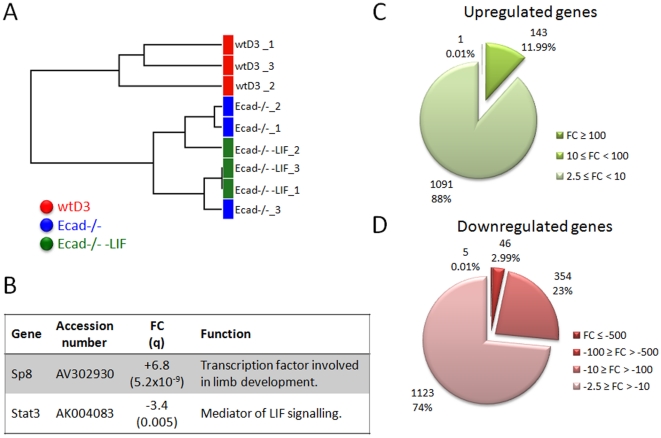
Ecad-/- ES cell expression profile is distinct from wtD3 ES cells irrespective of LIF-supplementation. RNA microarray analysis of gene expression was performed on wtD3 and Ecad-/- ES cells cultured in standard conditions in the presence of LIF and Ecad-/- ES cells cultured for 12 days in the absence of LIF. RNA was collected in three independent experiments for each cell line/condition. **A.** Dendrogram demonstrating clustering analysis of the microarray data for wtD3 (red), Ecad-/- (blue) and Ecad-/- -LIF (green) ES cells. **B.** Table showing the two genes that exhibited differential expression between Ecad-/- ES cells cultured in the presence and absence of LIF. Results were deemed significantly different if the q value was <0.05 and fold change ≥2.50. **C.** Analysis of fold change values of genes upregulated in Ecad-/- ES cells compared to wtD3 ES cells. Upregulated genes were fewer in number and fold change absolute values were lower in comparison to downregulated genes. 88% of the genes showed FC between 2.5 and 10, 11.99% had a FC between 10 and 100 and only 1 (0.01%) exhibited FC>100 **D.** Analysis of fold change values of genes downregulated in Ecad-/- ES cells compared to wtD3 ES cells. The absolute value of FC of downregulated genes was higher than the upregulated ones. 0.01% of genes had a FC below -500, 2.99% between -500 and -100, 23% exhibited FC between -100 and -10 whilst the larger proportion (73%) showed FC between -10 and -2.5. Data published in ArrayExpress E-MEXP-2836.

We have previously shown that Ecad-/- ES cells maintain expression of the pluripotency markers Nanog, Sox2 and Oct3/4 via Activin/Nodal signalling pathways in FBS-containing medium, irrespective of LIF supplementation [17]. Therefore, to determine the effect of LIF on these cells we compared gene expression profiles of Ecad-/- ES cells cultured in the presence or absence of LIF (ArrayExpress E-MEXP-2836). PCA of the microarray data confirmed the quality of the triplicate samples (Figure S1) and hierarchical clustering analysis identified that the main variation within the data sets was between wtD3 and Ecad-/- ES cells (Figure 1A). No clustering could differentiate between Ecad-/- ES cells grown in the presence and absence of LIF and any variation among these samples can be classified within the normal biological variance. These results suggest that LIF has little or no effect on gene expression profiles of Ecad-/- ES cells despite the LIF receptor and gp130 being localised at the cell surface (KH, unpublished data). Threshold levels of q<0.05 and fold-change (FC) ≥2.50 were chosen to determine statistical significance between transcript levels. Only 2 genes among over 45,000 probes were found to be statistically significant in Ecad-/- ES cells grown with or without LIF (Figure 1B). The trans-acting transcription factor 8 (Sp8) showed a 6.2-fold increase in the absence of LIF whereas the signal transducer and activator of transcription 3 (Stat3) was downregulated 3.4-fold. This observation suggests that Stat3 and Sp8 transcripts are likely to be regulated by LIF independently of E-cadherin and that LIF-mediated Stat3 activation is dispensable for Ecad-/- ES cell pluripotency.

### wtD3 and Ecad-/- ES cells exhibit distinct gene expression profiles

Comparison of transcript expression in wtD3 and Ecad-/- ES cells cultured in the presence of LIF revealed 2768 probes, representing 2265 genes, which were differentially expressed. Ecad-/- ES cells showed a slight bias towards downregulation of genes compared to wtD3 ES cells (55% downregulated in the former). Analysis of the magnitude of fold-change in gene expression revealed that the majority of upregulated genes in Ecad-/- ES cells fell between 2.5 and 10 fold-change (88% of total upregulated genes) whilst 12% were between 10 and 100FC (Figure 1C). Only 1 gene (Serpina3m, representing 0.01%) resulted in a positive fold-change above 100 (+165FC). Amongst the twenty most upregulated genes (Table 1), six were known glycoproteins and two were members of the Serpin family (a3m and b9), which are peptidase inhibitors with anti-apoptotic activity. With respect to downregulated genes in Ecad-/- ES cells, 74% exhibited a fold-change of -2.5 to -10, 23% showed a fold-change between -10 and -100 and 3% (46 genes) showed over 100-fold decreased transcript expression (Figure 1D). Amongst the twenty most downregulated genes (Table 2), 5 genes exhibited over 500-fold change and were mainly associated with gene expression regulation and transcription factor activity, such as Pycard. Overall, this data shows that inhibition of E-cadherin results in significant global gene transcript alterations, suggesting that E-cadherin plays a critical role in regulating the transcriptional phenotype of mES cells.

**Table 1:** List of the top 20 upregulated transcripts in Ecad-/- ES cells.

Gene	Accession No.	Common Name	FC	q value
**Serpina3m**	BC011158	Serine (or cysteine) peptidase inhibitor, clade A, member 3M	+165	<0.0001
**Cd59a**	NM_007652	CD59a antigen	+66	<0.0001
**Btla**	BM240873	B and T lymphocyte associated	+64	<0.0001
**Nefm**	NM_008691	Neurofilament, medium polypeptide	+62	<0.0001
**Serpinb9**	NM_009256	Serine (or cysteine) peptidase inhibitor, clade B, member 9	+61	<0.0001
**Slc39a8**	NM_026228	Solute carrier family 39 (metal ion transporter), member 8	+55	<0.0001
**Fgf5**	AV240088	Fibroblast growth factor 5	+52	<0.0001
**Psors1c2**	NM_020576	Psoriasis susceptibility 1 candidate 2 (human)	+50	<0.0001
**BC064078**	AW493518	cDNA sequence BC064078	+46	<0.0001
**Epha1**	NM_023580	Eph receptor A1	+40	<0.0001
**Irgm**	NM_008326	Immunity-related GTPase family, M	+38	0.001
**--**	W45978	cDNA clone IMAGE:1328649	+38	0.002
**Fst**	NM_008046	Follistatin	+38	<0.0001
**Il33**	NM_133775	Interleukin 33	+36	<0.0001
**Mfap3l**	AK017269	Microfibrillar-associated protein 3-like	+35	0.007
**Chst1**	NM_023850	Carbohydrate (keratan sulfate Gal-6) sulfotransferase 1	+34	0.001
**Galr2**	NM_010254	Galanin receptor 2	+31	<0.0001
**Gpr37**	BQ175510	G protein-coupled receptor 37	+29	<0.0001
**Cd44**	M27130	CD44 antigen	+28	<0.0001
**Cd59a**	AK005507	CD59a antigen	+27	<0.0001

(FC = fold-change).

**Table 2:** List of the top 20 downregulated transcripts in Ecad-/- ES cells.

Gene	Accession No.	Common Name	FC	q value
**EG653016**	AV101904	Predicted gene	−752	<0.0001
**4930517K11Rik**	AK005645	RIKEN cDNA	−712	<0.0001
**Esrrb**	AV333667	Estrogen related receptor, beta	−579	<0.0001
**Inhbb**	BB253137	Inhibin beta-B	−546	<0.0001
**Laptm5**	BB218107	lysosomal-associated protein transmembrane 5	−527	<0.0001
**Nr0b1/Dax1**	NM_007430	Nuclear receptor subfamily 0, group B, member 1	−450	<0.0001
**Pycard**	BG084230	PYD and CARD domain containing	−449	<0.0001
**Lgals3**	X16834	Lectin, galactose binding, soluble 3	−445	<0.0001
**Calml4**	AY061807	Calmodulin-like 4	−402	<0.0001
**Zfp42/Rex1**	NM_009556	Zinc finger protein 42	−353	<0.0001
**Slc38a4**	AK003626	Solute carrier family 38, member 4	−310	<0.0001
**Rnf17**	AV225034	Ring finger protein 17	−286	<0.0001
**Mras**	AB004879	Muscle and microspikes RAS	−280	<0.0001
**LOC245128**	AV099404	Similar to solute carrier family 7, member 3	−266	<0.0001
**Lrrc34**	AK005720	Leucine rich repeat containing 34	−260	<0.0001
**Slc38a4**	NM_027052	Solute carrier family 38, member 4	−259	<0.0001
**2410146L05Rik**	BB702364	RIKEN cDNA 2410146L05 gene	−256	<0.0001
**Tgfbi**	BB533460	Transforming growth factor, beta induced	−255	<0.0001
**--**	BG071670	Transcribed locus	−252	<0.0001
**Dppa5**	NM_025274	Developmental pluripotency associated 5	−247	<0.0001

(FC = fold-change).

**Figure 2:**
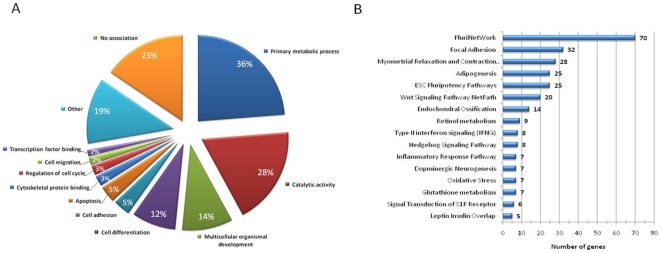
Gene Ontology and G0-Elite analysis. Pie chart showing the gene ontology terms that are most represented in the microarray analysis comparing wtD3 and Ecad-/- ES cells (**A**). Besides terms related to loss of cell adhesion (cell migration and cytoskeleton protein binding), the most represented genes belong to general biological processes such as primary metabolic processes (36%; representing 807 genes) and catalytic activity (28%; 625 genes). A large number of genes are also associated with cell differentiation (12%; 279 genes) and multicellular organismal development (14%; 320 genes). These results suggest that E-cadherin mediated cell-cell adhesion does not solely control the migratory ability of the cell but affects more general aspects of cellular biology. The numbers inside each pie slice show the percentage of total differentially expressed genes. **B**. G0-Elite analysis of Wikipathways. Networks associated with pluripotency are highly represented.

**Figure 3:**
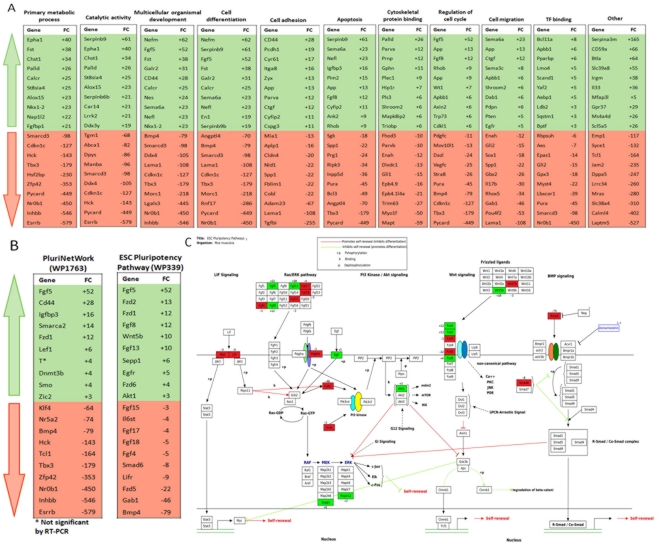
List of the top 10 upregulated and downregulated genes represented in the Gene Ontology analysis. List of the top 10 up-regulated (green) and down-regulated (red) genes (**A**) in each of the GO terms shown in Figure 2A. **B.** List of the top 10 up-regulated (green) and down-regulated (red) genes in the PluriNetWork and ESC Pluripotency pathways shown in Figure 2B. **C.** Network of transcripts associated with the ESC Pluripotency Pathway. Transcripts exhibiting altered expression in Ecad-/- ES cells are highlighted: upregulated transcripts are shown in green and downregulated transcripts shown in red (with fold-change indicated by the number). Reproduced with kind permission from WikiPathways (http://creativecommons.org/licenses/by/3.0/).

Gene Ontology (GO) (Figures 2A and 3A), KEGG pathway (Figure S2 and Table S3) and WikiPathway analysis (Figure 2B and 3B) of the 2265 differentially expressed genes were performed using the web-based software DAVID and GO-Elite. The GO analysis revealed that depletion of E-cadherin at the cell surface of mouse ES cells did not exclusively affect adhesion-associated transcripts (e.g. cell adhesion, cell migration) but had a wider transcriptional impact as shown by GO terms such as “primary metabolic process” and “catalytic activity”. The ten most up- and down-regulated genes in Ecad-/- ES cells in each of the GO terms (compared to wtD3) are shown in Figure 3A. The “primary metabolic process” group contained 807 genes representing 36% of the total gene list. Within this group, we found alterations in transcript expression of regulators of transcription (Esrrb, Nr0b1, Zfp42), signalling transducer/regulator molecules (Inhbb, Calcr, Fst) as well as genes related to protein phosphorylation (Epha1) and degradation (Pycard). Many of these genes appeared in other more specific GO categories (Figure 3A). Alterations in genes associated with catalytic activities included kinases (Hck, Epha1), hydrolases (Ddx4) and transferases involved in post-translational modifications, such as sulfotransferases (Chst1) and sialyltransferases (St8sia4). 14% of the transcript alterations were related to “multicellular organismal development” and 12% to “cell differentiation”, comprising genes involved in sex determination (Nr0b1, Morc1, Fst and Rnf17) and neuronal development (Nefm, Galr2), amongst others. Ecad-/- ES cells also exhibited down-regulation of genes associated with maintenance of ES cell pluripotency such as Nr0b1 and Tbx3 and up-regulation of proliferative genes (e.g. Fgf-5). The alteration in gene transcripts involved in regulation of cell cycle and apoptosis confirm our previous observations that Ecad-/- ES cells exhibit nearly 2-fold increased proliferation compared to wtD3 ES cells (Figure S3A) [17]. Cell cycle analysis did not reveal any significant differences between the proportion of cells in G1, S or G2 phase between wtD3 and Ecad-/- ES cells (Figure S3B), suggesting that increased proliferation of Ecad-/- ES cells is not due to altered cell cycle kinetics. Approximately 23% of the genes were not associated with any GO term and 19% were associated with GO groups containing less than 10 genes. The analysis of the pathways with the most enriched number of terms using the KEGG database confirmed the influence of E-cadherin on primary metabolic processes (Figure 2A). Six of the 20 terms (30%) were associated with metabolic pathways, in particular of carbohydrates (mmu00520 and mmu00051), lipid (mmu565 and mmu00071) and amino acids (mmu00310 and mmu00480) (Figure S2 and Table S3). Three terms were associated with cell communication and adhesion (mmu04510, mmu04530 and mmu04540) and one with the interaction of the cell with the extra-cellular matrix (mmu04512). These terms may reflect the influence of E-cadherin on cell adhesion and the single cell morphology observed in Ecad-/- ES cells.

G0-Elite software analysis identified altered pathways in Ecad-/- ES cells associated with embryonic stem cell pluripotency (Figures 2B and 3B). The PluriNetWork (WP1763), which comprises an intricate network of genes and mechanisms underlying mouse pluripotency, exhibited 70 altered transcripts (Figure 2B). The top 10 up- and down-regulated transcripts associated with the PluriNetWork and ES pluripotency pathways are shown in Figure 3B. A detailed analysis of transcript alterations associated with the mouse ES cell pluripotency network is shown in Figure 3C. Alterations in transcript expression were observed in a range of pluripotency-associated signalling pathways including LIF, Ras/Erk, FGF, PI3K, and BMPs (Figure 3C). Whilst transcripts encoding the LIF receptor and its complex partner gp130 (Il6st) were down-regulated, both proteins are present at the cell surface of Ecad-/- ES cells (KH, data not shown). This suggests that the alteration in pluripotency pathways in Ecad-/- ES cells is not a result of absence of these receptors at the cell surface. BMP4 encoding transcripts were decreased 79-fold compared to wtD3 ES cells and this may reflect the dependency of Ecad-/- ES cells on Activin/Nodal signalling, rather than LIF/BMP [17]. However, it should be noted that Ecad-/- ES cells are able to maintain pluripotency in LIF/BMP-supplemented medium in the absence of Activin/Nodal, suggesting that an alteration in pluripotency pathway hierarchy occurs in these cells, rather than an irreversible switch from LIF/BMP to the Activin/Nodal pathways [17]. We have previously demonstrated that Ecad-/- ES cells exhibit increased proliferation via FGF2 [17]. Although neither FGF2 nor its receptor (FGFR1) exhibited altered transcript expression in Ecad-/- ES cells, different ligands of the same family (e.g. FGF5) showed modified expression levels. Whilst the ES cell pluripotency transcript analyses reflect our previous observation that the LIF/BMP pathways are altered in Ecad-/- ES cells they do not, frustratingly, provide a definitive explanation for the dependence of these cells on Activin/Nodal and FGF2 signalling. However, up-regulation of the natural inhibitor of Activin A, Follistatin (FC  =  +38, Table 1), provides circumstantial evidence for the altered regulation of the Activin/Nodal pathway in Ecad-/- ES cells. Overall, these results show that Ecad-/- ES cells exhibit a distinct gene expression profile compared to wtD3, with variations associated with a large variety of biological processes, including alterations in pluripotency pathways.

### The Ecad-/- ES cell transcriptome exhibits more similarity with the epiblast-derived stem cell transcriptome than wtES cells

**Figure 4:**
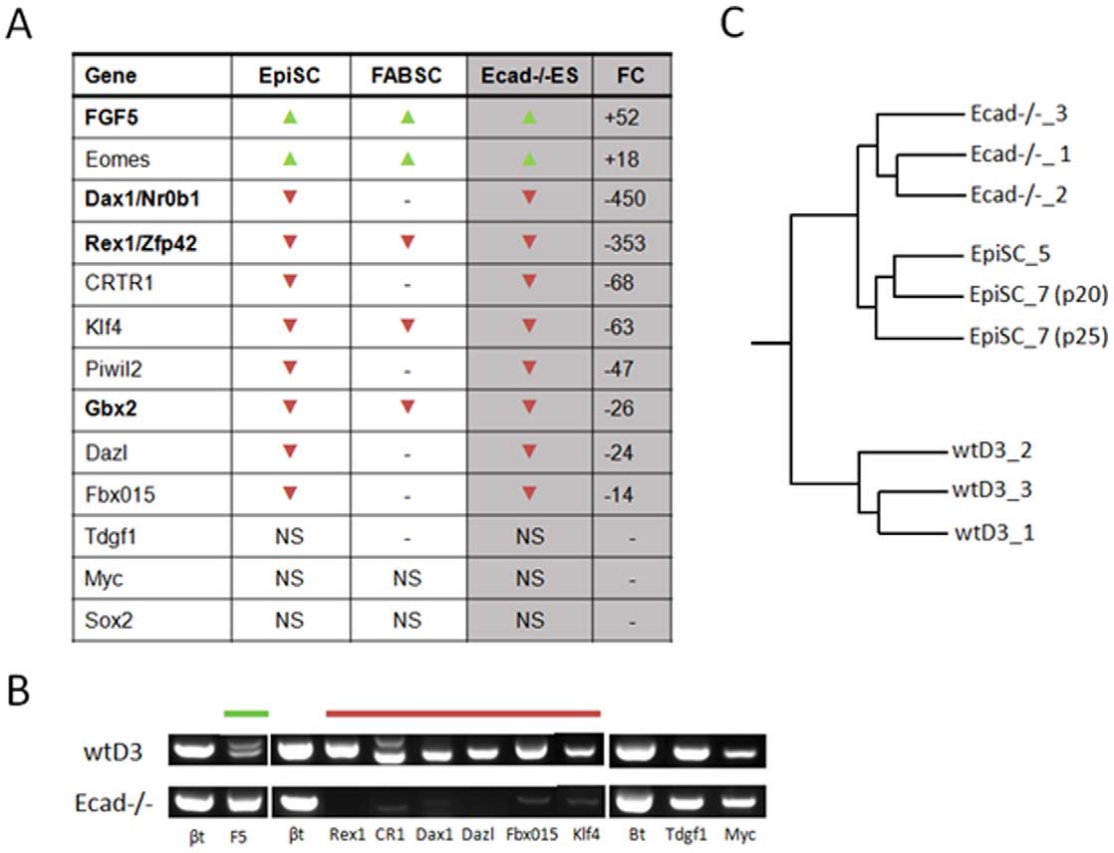
Ecad-/- ES cells show more transcriptional similarities to EpiS cells than to the parental wtES cell line. Table showing genes with similar expression patterns in Ecad-/-, EpiSC and FABS cells compared to wtES cells (**A**). The last column of the table shows the fold-change (FC) calculated in our microarray analysis. NS  =  Not Significant. **B.** Validation of the microarray data by RT-PCR. FGF-5 (upregulated), Rex1, CRTR1, Dax1, Dazl, Fbx015 and Klf4 (downregulated) and the unchanged genes Tdgf1 and Myc confirmed the microarray results. **C.** Dendrogram generated by hierarchical clustering of the microarray data for wtD3, Ecad-/- and EpiSCs (Brons et al. (2007) [28]). Data for EpiS and FABS cells were taken from Brons et al. (2007) [28] and Chou et al. (2008) [16].

Pluripotent cells derived from the epiblast tissue of embryos (EpiS cells) [28], [31] have been described to self-renew via the Activin/Nodal pathway using FGF as a proliferative factor in a similar manner to Ecad-/- ES cells, previously described by our group [17]. Therefore, we investigated the similarities in transcript expression between Ecad-/- ES cells and EpiS cells according to available published data [28], [31]. We also compared the transcript expression of FABS cells, stem cells derived from mouse ICM using a chemically defined medium containing FGF2, Activin, BIO and a LIFR neutralising antibody [16]. Up-regulation of Fgf-5 and down-regulation of Zfp42/Rex1 and Nr0b1 have been described as signature markers of EpiS cells [28], [31] and this was shared between all cell lines in addition to increased levels of Eomes transcripts (Figures 4A and 4B; Figure S5). In addition, transcripts encoding CRTR1, Klf4, Piwil2, Gbx2, Dazl and Fbx015 were all down-regulated in Ecad-/- and EpiS cells (Figures 4A and 4B; Figure S5). Tdgf1, Myc and Sox2 showed no gene expression changes in the target cell lines (Figure 4A and 4B). Despite the similarity in core gene transcript expression between Ecad-/- and EpiS cells, hierarchical analysis of the data sets demonstrated that the cell lines exhibit distinct transcript expression patterns (Figure 4C). However, the Ecad-/- ES cell transcriptome exhibits more similarity with EpiS cells than wtES cells, demonstrating that Ecad-/- ES cells are more closely related to an epiblast phenotype than that of the ICM. Tables S4, S5, S6 and S7 compare the top 20 up- and down-regulated genes in Ecad-/- and EpiSCs to further illustrate the similarities and differences in transcript expression between these two cell lines. For example, whilst there is a significant degree of similarity in the trend of downregulated transcripts, this is less apparent in upregulated transcripts. Overall, these results demonstrate that whilst Ecad-/- ES cells share similar transcriptional traits with EpiS cells they are a distinct cell type with a unique transcriptional signature.

### Ecad-/- ES cells do not exhibit endogenous β-catenin/TCF activity

Since E-cadherin can sequester β-catenin at the cell surface, potentially regulating the amount of β-catenin available for canonical Wnt transactivation, we decided to investigate the levels of β-catenin-mediated nuclear activity in Ecad-/- ES cells. Whilst our previous data suggested no fundamental role for canonical Wnt pathway in the maintenance of ES cell pluripotency [17] we did not assess global transcript alterations in Ecad-/- ES cells nor the impact of β-catenin in regulating non-pluripotency associated transcripts. One of the most represented signalling pathways in the Ecad-/- ES cell array highlighted by KEGG and WikiPathway analysis was the Wnt pathway (Figures 2B, S4 and Tables 2A and S3). Analysis of gene transcript changes associated with the Wnt pathway demonstrated alteration in transcripts encoding ligands, receptors and antagonists (Table 3 and Figure S4). Whilst almost every aspect of the Wnt-pathway is represented, some elements are upregulated (e.g. Fzd1, 2, Wnt5b, Wnt8a, TCF7, LEF1) whilst others are downregulated (e.g. Fzd5, Wnt7b, TCF15). In addition, some elements of the β-catenin degradation pathway are increased (e.g. Axin2, Snk1d) whereas others are decreased (e.g. Ppp2r5a). This data suggests that E-cadherin functions to both positively- and negatively-regulate the Wnt pathway and associated transcripts. Analysis of β-catenin/TCF target genes revealed that of the 40 genes assessed 73% exhibited no significant alteration in Ecad-/- compared to wtES cells (Table 4). Whilst six β-catenin-transactivated transcripts were upregulated in Ecad-/- ES cells, four were downregulated, suggesting divergent β-catenin/TCF-induced gene transactivation.

**Table 3:** Gene transcripts associated with Wnt signalling pathway.

Accession number	Gene	FC	Accession number	Gene	FC
**Ligands**			**Nuclear activity**		
NM_009525	Wnt5b	8.31	AI323642	Tcf7	2.63
W29605	Wnt7b	−3.36	NM_009328	Tcf15	−8.89
NM_009290	Wnt8a	21.59	NM_010703	Lef1	5.90
**Receptors**			BG073323	Chd8	−4.15
BB259670	Fzd1	11.64	**Other**		
BB371406	Fzd2	13.25	AB036749	Porcn	−7.85
AU043193	Fzd3	−2.62	BB353860	Frat2	−3.19
NM_022721	Fzd5	−22.22	BC006875	Prkx	2.89
NM_008056	Fzd6	3.54	**Non-canonical Wnt pathway**		
**Antagonist**			NM_008874	Plcb3	−4.85
BB497685	Sfrp1	−2.52	BM730668	Plcb4	−4.10
NM_009144	Sfrp2	7.91	AW490258	Camk2a	−2.85
**β-Catenin degradation machinery**			NM_007595	Camk2b	−3.06
BB398993	Axin2	6.16	BC022643	Prickle1	23.18
NM_139059	Csnk1d	2.89			
BB325197	Ppp2r5a	−4.83			

**Table 4:** β-Catenin/TCF-regulated transcripts altered in Ecad-/- ES cells

Accession number	Gene	FC	Accession number	Gene	FC
M36277	Mycn	NS	NM_010919	Nkx2-2	NS
NM_007865	Dll1	NS	NM_018865	Wisp1	NS
NM_008238	Foxn1	NS	NM_016873	Wisp2	NS
NM_011427	Snai1	NS	X75557	Mrpplf3	NS
BM234360	Fn1	−6.12	U25633	Emp1	−116.90
BQ176915	Isl1	NS	NM_010512	Igf1	NS
NM_009291	Stra6	NS	NM_009506	Vegfc	−20.48
NM_009404	Tnfsf9	NS	NM_011075	Abcb1b	NS
NM_010110	Efnb1	NS	NM_031168	Il6	NS
BC003264	Enpp2	16.83	BC019986	Cdx1	NS
NM_012043	Islr	NS	NM_007674	Cdx4	NS
NM_010809	Mmp3	NS	NM_009144	Sfrp2	7.91
NM_009309	T*	4.35	AB006320	Pitx2	NS
NM_007541	Bglap1-2	NS	U03425	Egfr	4.98
BC019986	Cdx1	NS	NM_010099	Eda	NS
M94967	Ptgs2	NS	BC021411	Ovol1	NS
NM_008393	Irx3	NS	AV359819	Jag1	NS**
D83144	Six3	NS	NM_009877	Cdkn2a	NS
NM_010896	Neurog1	NS	BB709552	Fgf4	−4.76
NM_022435	Sp5	6.79	NM_013869	Tnfrsf19	3.33

*Not significant by RT-PCR.

**FC = 2.3, q<0.05. NS according to our threshold of FC≥2.50.

**Figure 5:**
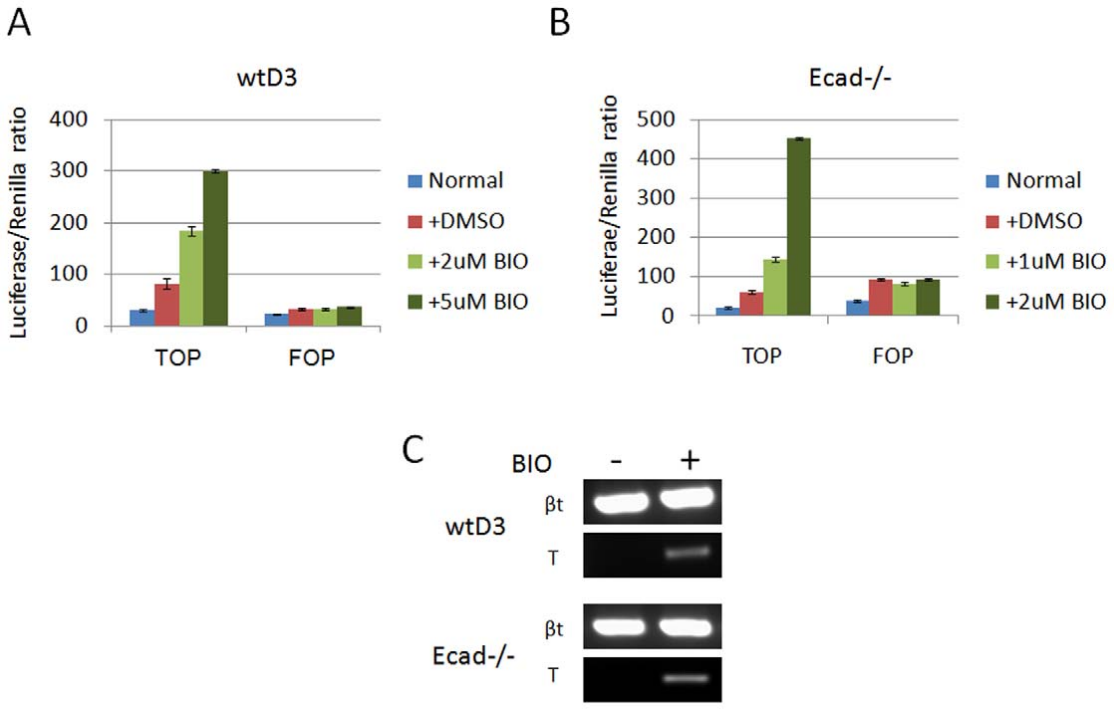
Ecad-/- ES cells show low basal Wnt activity similar to wtD3 ES cells. TOPflash and FOPflash reporter plasmid expression (**A**) in wtD3 ES cells cultured in LIF-supplemented standard medium (blue bar), DMSO control (red bar), 1 µM BIO (light green bar) and 2 µM BIO (dark green bar). **B.** TOPflash and FOPflash reporter plasmid expression in Ecad-/- ES cells cultured in LIF-supplemented standard medium (blue bar), DMSO control (red bar), 1 µM BIO (light green bar) and 2 µM BIO (dark green bar). **C.** RT-PCR expression of Brachyury (T), a typical Wnt activity marker, in wtD3 and Ecad-/- ES cells grown in the presence (+) and absence (−) of 2 µM BIO. βt  =  β-tubulin (loading control). Results present mean ± SD of one single experiment. At least, three independent experiments were performed.

To determine β-catenin/TCF activity in Ecad-/- ES cells we investigated levels of β-catenin-mediated transactivation in Ecad-/- ES cells compared to wtD3 ES cells using the reporter plasmid TOPflash. TOPflash contains the firefly luciferase gene under the control of several repeats of the β-catenin/TCF promoter region, whilst the control plasmid FOPflash contains a mutated version of the same promoter. As previously reported [13], [14], wtD3 ES cells lacked β-catenin/TCF activity, as demonstrated by similar levels of luciferase activity of both TOPflash and FOPflash plasmids (Figure 5A, blue bars). Ecad-/- ES cells also exhibited very low levels of TOPflash expression in LIF-supplemented medium with a slightly higher level observed for FOPflash (Figure 5B, blue bars), demonstrating lack of β-catenin/TCF activity in these cells. To investigate the ability of the cells to activate β-catenin-mediated gene expression in response to Wnt-like stimuli, both wtD3 and Ecad-/- ES cells were treated with the small molecule BIO, an inhibitor of GSK3, or with equivalent volumes of DMSO (control). Whilst BIO is not a specific Wnt activator, the presence of β-catenin/TCF promoter repeats within the TOPflash vector, and mutated regions in the FOPflash vector, enable the specificity of β-catenin/TCF activity to be assessed. WtD3 ES cells showed a dose-dependent TOPflash response to BIO (Figure 5A, light and dark green bars) which was significantly higher than the DMSO control (Figure 5A, red bar). The mutated reporter plasmid, FOPflash, did not exhibit differential activation in wtD3 ES cells in the presence of either DMSO (Figure 5A, red bar) or BIO (Figure 5A, light and dark green bars). Ecad-/- ES cells also exhibited a dose-dependent increase in TOPflash response to BIO (Figure 5B, light and dark green bars) compared to the control DMSO (Figure 5B, red bar). FOPflash did not exhibit differential activity in Ecad-/- ES cells in the presence of either DMSO or BIO (Figure 5B, red and green bars).

To confirm the TOPflash analysis, we assessed the expression of the mesoderm marker Brachyury (T), which has been reported to be regulated via β-catenin/TCF transactivation [2]. Whilst Brachyury was not detected in wtD3 or Ecad-/- ES cells by RT-PCR, treatment of these cells with BIO resulted in upregulation of transcripts in both cell lines (Figure 5C). Similar results were also observed in Ecad-/- cells cultured with BIO in the absence of LIF (data not shown). These data demonstrate that Ecad-/- ES cells exhibit very low levels of β-catenin/TCF transactivation, similar to that observed in wtD3 ES cells. Overall, these results suggest that loss of E-cadherin in ES cells does not induce β-catenin/TCF transactivation, in contrast to previously published data [2]. Moreover, the data also suggests that a pool of β-catenin protein is present within Ecad-/- ES cells which is able to respond to Wnt stimuli, as demonstrated by dose-dependent BIO activation.

### Ecad-/- ES cells exhibit reduced β-catenin protein levels compared to wtD3 ES cells

**Figure 6:**
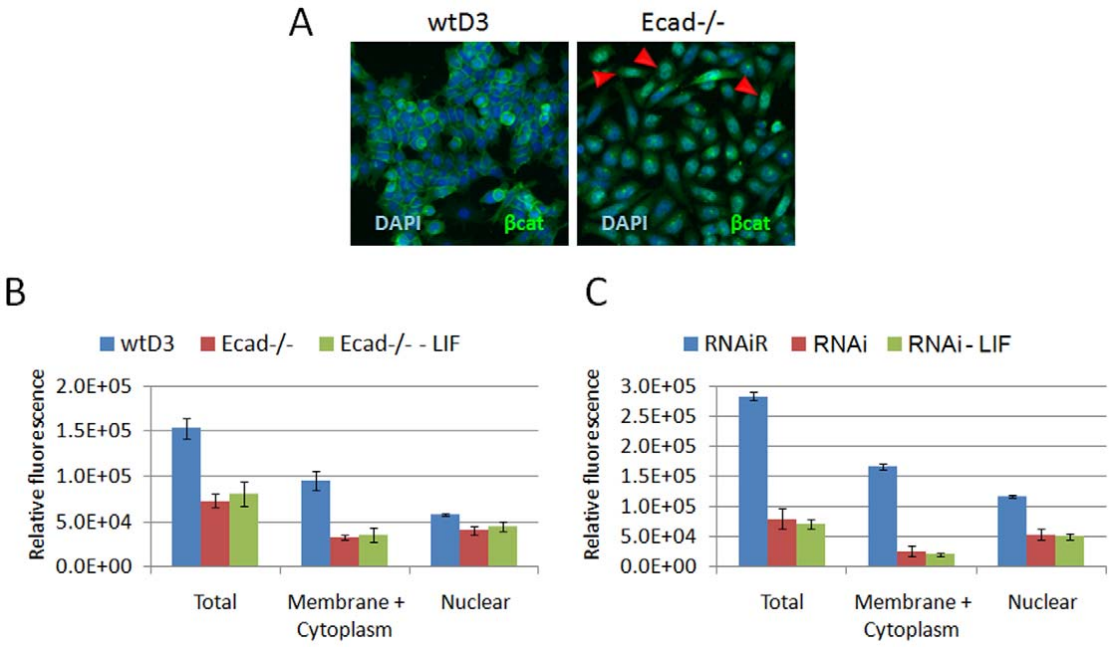
E-cadherin-depleted ES cells exhibit lower β-catenin protein levels compared to parental ES cells. Immunoflurescence microscopy images of β-catenin protein expression in wtD3 and Ecad-/- ES cells (**A**). Red arrows show nuclear localisation of β-catenin protein in Ecad-/- ES cells. **B.** Total and compartmental quantification of β-Catenin protein in wtD3 (blue) and Ecad-/- (red) ES cells cultured in LIF and Ecad-/- ES cells cultured in the absence of LIF (green). Error bars represent standard error of the mean of three independent experiments. **C.** wtES cells were transfected with an E-cadherin targeting hairpin-loop RNAi vector (RNAi) to derive clones lacking E-cadherin protein expression [17]. E-cadherin expression in the RNAi clone was subsequently induced by inhibition of the RNAi vector (RNAiR [17]). Total and compartmental quantification of β-catenin protein expression in RNAiR (blue) and RNAi (red) ES cells cultured in the presence of LIF and RNAi ES cells cultured in the absence of LIF (green) are shown. Similar to Ecad-/- ES cells, RNAi ES cells showed lower level of total β-catenin proteins compared to the rescued cell line RNAiR (red and blue, respectively). Error bars represent standard error of the mean of three independent experiments.

Since Ecad-/- ES cells exhibited negligible β-catenin/TCF transactivation, we investigated the levels of compartmentalised β-catenin protein in these cells compared to wtD3 ES cells (Figure 6). β-catenin protein was detected in wtD3 and Ecad-/- ES cells using immunofluorescent microscopy analysis (Figure 6A) and quantified using the Arrayscan compartmental analysis algorithm by Imagen Biotech (Manchester, UK). Ecad-/- ES cells showed approximately 50% lower levels of total β-catenin protein compared to wtD3 ES cells (Figure 6B, red and blue histogram, respectively). In wtD3 ES cells, β-catenin was mainly localised at the cell membrane (Figure 6A and 6B, blue histogram). In contrast, Ecad-/- ES cells grown in standard LIF medium exhibited diffuse β-catenin protein throughout the cytoplasm with some cells showing nuclear localisation (shown by arrows in Figure 6A). Quantification of the protein expression levels revealed similar distribution of the protein between the membrane/cytoplasm and the nucleus of Ecad-/- ES cells (Figure 6B, red bars). No significant differences in β-catenin localisation or expression were observed between Ecad-/- ES cells cultured in the presence or absence of LIF (Figure 6B, red and green histogram, respectively). Overall, this data shows that Ecad-/- ES cells exhibit significantly decreased levels of membrane/cytoplasmic β-catenin compared to wtD3 ES cells whereas nuclear β-catenin levels are similar in both cell lines. To confirm that the levels of β-catenin protein are affected by E-cadherin, we performed the same quantification assay in EcadRNAi ES cells, where E-cadherin protein expression is inhibited by siRNA plasmids and in EcadRNAiR, a rescued cell line where the siRNA effect is silenced thus restoring normal levels of E-cadherin expression [17]. The assay showed significantly lower levels of total β-catenin protein in EcadRNAi cells compared to EcadRNAiR cells (Figure 6C, red and blue bars, respectively), whereas no significant difference in expression levels were observed in EcadRNAi cells cultured in the presence or absence of LIF (Figure 6C, red and green bars, respectively). In EcadRNAi cells, grown in either the presence or absence of LIF, β-catenin was mainly localised within the nucleus (Figure 6C, red and green bars, respectively). In contrast, the rescued cell line EcadRNAiR showed high levels of β-catenin protein in the membrane+cytoplasm compartment (Figure 6C, blue histogram). These results suggest that E-cadherin expression may act as positive regulator of total β-catenin protein levels in mouse ES cells.

## Discussion

We have previously shown that Ecad-/- ES cells exhibit altered response to exogenous factors, maintaining pluripotency marker expression via the Activin/Nodal pathways and optimal self-renewal via FGF2 [17]. In this study, we have examined and compared the gene expression profiles of wtD3 and Ecad-/- ES cells cultured in standard LIF conditions and those of Ecad-/- ES cells cultured in the absence of LIF by Affymetrix microarray analysis.

Our data show that LIF exerts limited influence on the gene expression profile of Ecad-/- ES cells as demonstrated by the near identical transcript profiles obtained from these cells cultured in the presence or absence of LIF. Of over 45,000 probes assessed, only 2 genes showed statistically significant differences in transcript expression, Sp8 and Stat3. The transcription factor Sp8, which was upregulated 6.2-fold in the absence of LIF, has been reported to be critical during the formation of the apical ectodermal ridge for the correct outgrowth of limbs and proper closure of the cranial box. Research has suggested that Sp8 works downstream of the Wnt3, Fgf and BmpR cascades that regulate this phase of embryo development [32], [33]. However, no function in ES cells has been attributed to Sp8. Stat3, which showed 3.4-fold decreased expression in Ecad-/- ES cells in the absence of LIF, can be activated by various cytokines and is responsible for the transduction of LIF-mediated signals in the maintenance of pluripotency in mES cells. Interestingly, recent studies suggest the presence of positive feedback between LIF activity and Stat3 transcript levels [34], [35], [36]. Therefore, Stat3 downregulation in Ecad-/- ES cultured in the absence of LIF suggests that these cells exhibit a minimal response to LIF. This hypothesis is corroborated by the down-regulation of LIFR and gp130 (Ilst6) even in the presence of LIF within the culture media.

The previously observed LIF-independent pluripotency of Ecad-/- ES cells was confirmed by the down-regulation of Socs3, Tbx3 and Klf4, in the presence or absence of LIF. Tbx3 and Klf4 have recently been identified as down-stream effectors of two parallel circuitries of the LIF pathway [35] whilst Socs3 has been reported as a direct target of Stat3 [37]. As suggested by Niwa and colleagues [35], the function of certain genes, such as Tbx3 and Klf4, might be strictly cellular context-related and different gene sets might be associated with various extracellular signals to converge in the maintenance of the same core of transcription factors (e.g. Nanog, Sox2, Oct3/4). Recent studies have reported the ability of the Smad2/3 pathway, activated via activins, to sustain Nanog expression in EpiS cells [38]. We have previously demonstrated that inhibition of this pathway in Ecad-/- ES cells causes rapid differentiation of the cells with loss of Nanog expression [17]. We have also observed that Ecad-/- ES cells can be cultured in LIF/BMP medium and that wtES cells exhibit reversible Activin/Nodal-dependent pluripotency when treated with an E-cadherin inhibiting peptide [17]. This suggests that the maintenance of pluripotency via extrinsic factors is likely to be more complex than previously described, with E-cadherin playing a pivotal role in regulating hierarchical signalling via the LIF/BMP and Activin/Nodal pathways. Whilst the core transcription factors associated with pluripotency are expressed in Ecad-/- ES cells, the pathways which regulate these factors are clearly different to wtES cells. For example, it is likely that Ecad-/- ES cells maintain pluripotency via Smad2/3-induced transactivation of target genes rather than the signalling networks currently attributed to mouse ES cells. It has been previously demonstrated that ES cells exhibit an innate ability for replication that does not appear to require intercellular stimulation and is intrinsically self-maintaining, akin to that observed in unicellular organisms [39]. Our results suggest that this pluripotent ‘ground state’ is unaltered in Ecad-/- ES cells and that the function of E-cadherin is to regulate extrinsic stimuli that converge to maintain expression of the core pluripotency-associated transcripts Oct3/4, Nanog and Sox2.

The observation of the similarities between Ecad-/- ES, EpiS and FABS cells raises interesting questions about the impact of E-cadherin on gene expression. Hierarchical analysis of the array data showed that Ecad-/- ES cells exhibit more similarities with EpiS cells than wtES cells. The question is whether, and how, some of the transcriptional similarities between Ecad-/- ES and EpiS/FABS cells are mediated by E-cadherin protein expression. Although E-cadherin protein levels in EpiS cells have not been described, a correlation has been reported in FABS cells [16]. FABS cells exhibited low levels of E-cadherin transcript and protein and showed low chimerism and teratoma formation ability together with limited proliferation when cultured in suspension. However, upon LIF/BMP4 stimulation, FABS cells up-regulated E-cadherin and showed improved chimerism ability and teratoma formation. These features were maintained upon reversal of the growth conditions to FABS cell media which was associated with the maintenance of E-cadherin expression.

It has been previously reported that Ecad-/- ES cells exhibit increased levels of brachyury transcripts and that this is achieved by β-catenin/TCF-induced gene transactivation [2], [40]. However, our analysis of Ecad-/- ES cells has failed to confirm these observations. The decreased expression of total β-catenin protein in both Ecad-/- and EcadRNAi ES cells does not correlate with β-catenin transcript expression in these cells, suggesting that E-cadherin may stabilise β-catenin protein levels in wtES cells. Although the nuclear to membrane/cytoplasmic ratio of β-catenin in Ecad-/- ES cells was higher than that observed in wtD3 ES cells, the quantity of protein localised within the nucleus was similar in both cell lines. These results suggest that the pool of β-catenin normally recruited at the cell surface by E-cadherin is almost entirely degraded and a new homeostatic equilibrium is achieved in E-cadherin-depleted cells, resulting in lower total β-catenin protein levels. This hypothesis is corroborated by the increased levels of β-catenin protein measured in the rescued EcadRNAiR ES cell line. Our data also support the hypothesis that β-catenin exists within cells in two discrete forms, one with high cell adhesion specificity and another for nuclear signalling and that these two forms are not completely interchangeable [41]. These results are also in accordance with the mathematical model presented by van Leeuwen and colleagues [42], which expanded previously developed models and predicted that E-cadherin expression levels and β-catenin nuclear levels are uncoupled.

Contrasting results were observed for the mesoderm marker Brachyury, whose expression has been shown to be regulated by the Wnt pathway via TCF/LEF binding sites within the promoter region [40]. Brachury mRNA was not detected in wtD3 or Ecad-/- ES cell lines by RT-PCR or qPCR but was detected upon treatment of the cells with BIO. However, the microarray analysis showed a statistically significant 4.3-fold increase in Brachyury transcripts in Ecad-/- ES cells compared to wtD3 ES cells (q = 0.04). These contrasting results might be explained by the different sensitivity of the two methods, with the microarray analysis able to detect differences even in very low transcriptionally active genes. This is corroborated by our qPCR analysis of Fgf5 and Eomes, where levels were found to be an order of magnitude lower than detected in the microarray, although the trends were similar. The low Brachyury transcript expression in our study is in contrast with Kemler and colleagues' initial characterisation of Ecad-/- ES cells, who detected Brachyury transcripts in Ecad-/- but not wtD3 ES cells [2]. However, this discrepancy is likely to be a result of different culture conditions used in these two studies. Our analysis was performed in ES cells cultured in ES cell screened FBS in the absence of a fibroblast feeder layer, which results in low Wnt activity in wt mES cells [13]. In contrast, Kemler and colleagues cultured their ES cells in buffalo rat liver (BRL)-conditioned medium which may contain higher levels of Wnt proteins or other exogenous factors which could promote brachyury expression in Ecad-/- ES cells.

In the comparison of gene expression profiles of wtD3 and Ecad-/- ES cells cultured under standard LIF conditions, we obtained more than 2200 genes exhibiting statistically significant difference in transcript expression and these were involved in various fundamental biological processes. Microarray data can be analysed according to the predominance of gene ontology terms. In our analysis, we considered GO terms of the Molecular Function (MF) and Biological Process (BP) branches up to level 5. The extent of GO terms associated with Ecad-/- ES cell transcript alterations is surprising and demonstrates that E-cadherin protein plays a major function in maintaining the transcriptional phenotype of mouse ES cells. Indeed, E-cadherin functions as both a positive- and negative-effector of transcripts associated with a breadth of cellular processes. Whilst further experimental analysis of the biological relevance of these transcript alterations is desirable, it does suggest that E-cadherin plays an important role in regulating numerous cellular processes that, as yet, have not been attributed to this protein. Since microarray analysis is a subjective process (for example, we have determined that fold-changes ≤2.50 and ≥2.50 are significant and only GO terms with at least 10 genes were included) it is likely that E-cadherin regulates many other cellular processes that do not meet our assessment criteria. We discuss transcript alterations of some genes associated within specific gene ontology terms in Text S1.

The most abundant term identified in the KEGG pathway analysis (Figure S2) was “pathways in cancer”, with 72 gene transcript alterations. Genes represented in this category are also associated with melanoma (mmu05218), prostate cancer (mmu05215), colorectal cancer (mmu05210), basal cell carcinoma (mmu05217), renal cell carcinoma (mmu05211) and glioma (mmu05214). When viewed in the context of metastatic transformation of epithelial cancer cells, with the proviso that the effects of E-cadherin in mES cells are mirrored in human epithelial cells, these observations may explain why loss of E-cadherin in epithelial tumours is associated with a more aggressive phenotype. Loss of E-cadherin is a common characteristic of aggressive epithelial tumours and is frequently associated with an EMT event, leading to upregulation of various MMPs and acquisition of a more motile phenotype, which increases the metastatic property of the cells [1]. However, our study suggests that E-cadherin might exert a much wider impact on the gene expression profile of cancer cells, affecting general biological processes of the cells as well as increased motility. In this study we have observed modified transcript levels of anti-apoptotic genes, alterations in the cellular metabolism and cell cycle. Such modifications, together with the altered response to growth factors present in the environment, might represent a significant survival and proliferative advantage for tumour cells [43].

### Conclusion

Our data show that loss of E-cadherin has effects not only confined to cellular adhesion but also on the general biology of ES cells. We have demonstrated that Ecad-/- ES cells exhibit gene expression profiles more similar to EpiS cells and FABS cells than to wt ES cells. However, they clearly present a unique gene expression profile. We have also shown that expression of E-cadherin influences the levels of total β-catenin protein as demonstrated by the decreased β-catenin levels in E-cadherin depleted cells (Ecad-/- and EcadRNAi). β-catenin protein in Ecad-/- ES cells is distributed equally between the cytoplasm and the nucleus. However, the nuclear fraction of β-catenin does not exhibit significant transactivity, suggesting that loss of E-cadherin alone is insufficient to induce β-catenin/TCF transactivation. Overall, these results suggest a much wider role for E-cadherin in regulating cellular homeostasis than is currently presumed and, following abrogation of this control, significant alterations in cellular phenotype occur that may then reflect the critical role of this protein in embryogenesis and the more aggressive phenotype of metastatic tumour cells.

## Supporting Information

Figure S1 **Principal component analysis map of the microarray data.** The graph shows that component 1 clearly distinguished between the data set of wtD3 samples (red) from those of the two Ecad-/- ES cell samples (with and without LIF, blue and green respectively). By contrast, no component was able to distinguish between Ecad-/- ES cells grown in the presence or absence of LIF.
(TIF)

Figure S2 **KEGG pathway analysis.** Pie chart showing the most represented KEGG pathway terms in the comparison between wtD3 and Ecad-/- ES cell transcripts. The most represented term is “pathways in cancers” while 6 terms are related to various metabolic processes, confirming the observation in the GO analysis. Four terms are related to cell adhesion, particularly focal adhesion, tight and gap junctions. Wnt pathway is the second most abundant signalling cascade after MAPK, followed by Hedgehog.
(TIF)

Figure S3 **Analysis of proliferation and cell cycle in wtD3 and Ecad-/- ES cells.** Proliferation of wtD3 and Ecad-/- ES cells was assessed over 5 days and cumulative viable cell numbers measured over this period (**A**). Cell cycle analysis of wtD3 and Ecad-/- ES cells cultured in the presence of LIF (**B**).
(TIF)

Figure S4 **Network of transcripts associated with the Wnt signalling pathway.** Transcripts exhibiting altered expression in Ecad-/- ES cells are highlighted: upregulated transcripts are shown in green and downregulated transcripts shown in red (with fold-change indicated by the number). Reproduced with kind permission from WikiPathways (http://creativecommons.org/licenses/by/3.0/).
(TIF)

Figure S5 **qPCR snd RT-PCR analysis of EpiSC-associated transcripts in Ecad-/- ES cells compared to wtD3 ES cells.** qPCR analysis of FGF5, Eomes, Gbx2, Blimp1, Lefty2, Stella/Dppa3, SSEA-1 and Nodal in Ecad-/- ES cells compared to wtD3 ES cells (**A**). This analysis confirms the results of the microarray data. **B.** RT-PCR analysis of Otx2, Pitx2, Nodal and Acvr2b demonstrating similar expression of these transcripts in Ecad-/- and wtD3 ES cells. (βt  =  β-tubulin, loading control).
(TIF)

Table S1 **Primer sequences for RT-PCR analysis.**
(DOC)

Table S2 **Primer sequences for qPCR analysis.**
(DOC)

Table S3 **Signalling pathways identified in the microarray analysis as exhibiting significant alterations in Ecad-/- compared to wtD3 ES cells.**
(DOC)

Table S4 **20 most upregulated probes in wtD3 vs Ecad-/- compared to wtES vs EpiSCs.** (FC = fold-change)
(DOC)

Table S5 **20 most downregulated probes in wtD3 vs Ecad-/- compared to wtES vs EpiSCs.**
(DOC)

Table S6 **20 most upregulated probes in wtES vs EpiSCs compared to wtD3 vs Ecad-/- ES cells.** (FC = fold-change)
(DOC)

Table S7 **20 most downregulated probes in wtES vs EpiSCs compared to wtD3 vs Ecad-/- ES cells.** (FC = fold-change)
(DOC)

Text S1 (DOCX)
