# Supplementary figures and images for: E-Cadherin Acts as a Regulator of Transcripts Associated with a Wide Range of Cellular Processes in Mouse Embryonic Stem Cells
Source: PLoS One. 2011 Jul 14;6(7):e21463. doi: 10.1371/journal.pone.0021463 (PMC3136471; doi:10.1371/journal.pone.0021463)

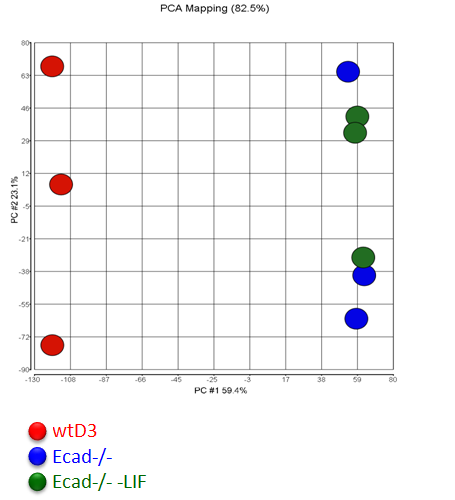

Supplement: Figure S1 — Principal component analysis map of the microarray data. The graph shows that component 1 clearly distinguished between the data set of wtD3 samples (red) from those of the two Ecad-/- ES cell samples (with and without LIF, blue and green respectively). By contrast, no component was able to distinguish between Ecad-/- ES cells grown in the presence or absence of LIF. (TIF) [file pone.0021463.s001.tif]

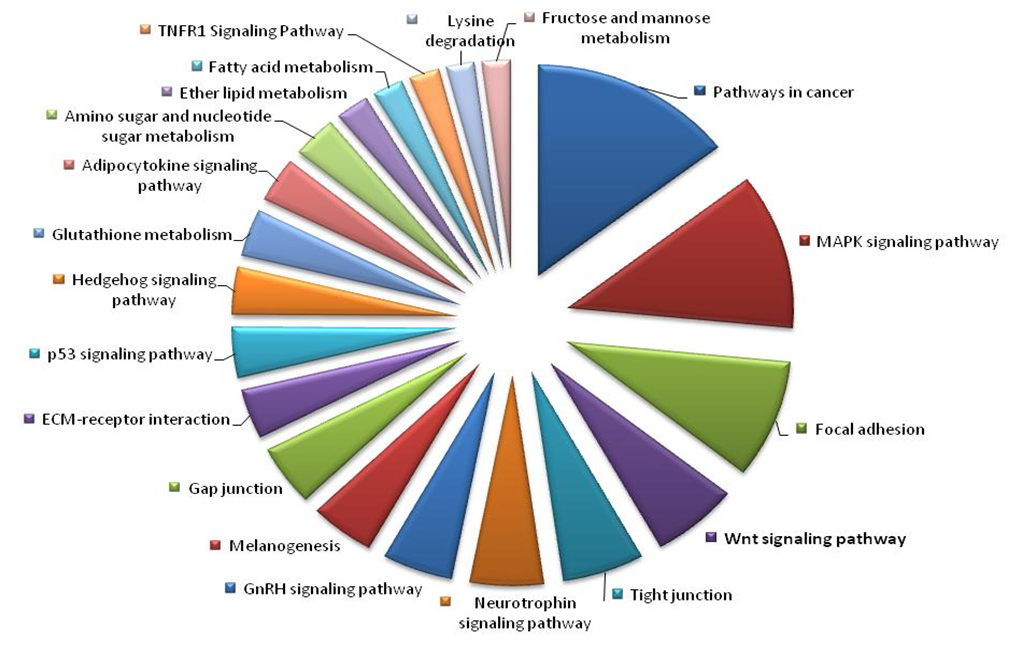

Supplement: Figure S2 — KEGG pathway analysis. Pie chart showing the most represented KEGG pathway terms in the comparison between wtD3 and Ecad-/- ES cell transcripts. The most represented term is “pathways in cancers” while 6 terms are related to various metabolic processes, confirming the observation in the GO analysis. Four terms are related to cell adhesion, particularly focal adhesion, tight and gap junctions. Wnt pathway is the second most abundant signalling cascade after MAPK, followed by Hedgehog. (TIF) [file pone.0021463.s002.tif]

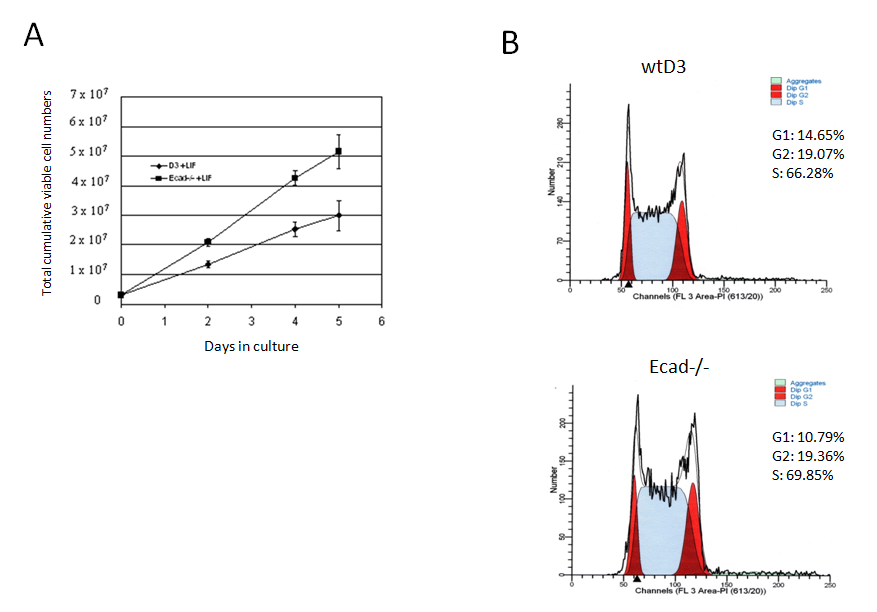

Supplement: Figure S3 — Analysis of proliferation and cell cycle in wtD3 and Ecad-/- ES cells. Proliferation of wtD3 and Ecad-/- ES cells was assessed over 5 days and cumulative viable cell numbers measured over this period (A). Cell cycle analysis of wtD3 and Ecad-/- ES cells cultured in the presence of LIF (B). (TIF) [file pone.0021463.s003.tif]

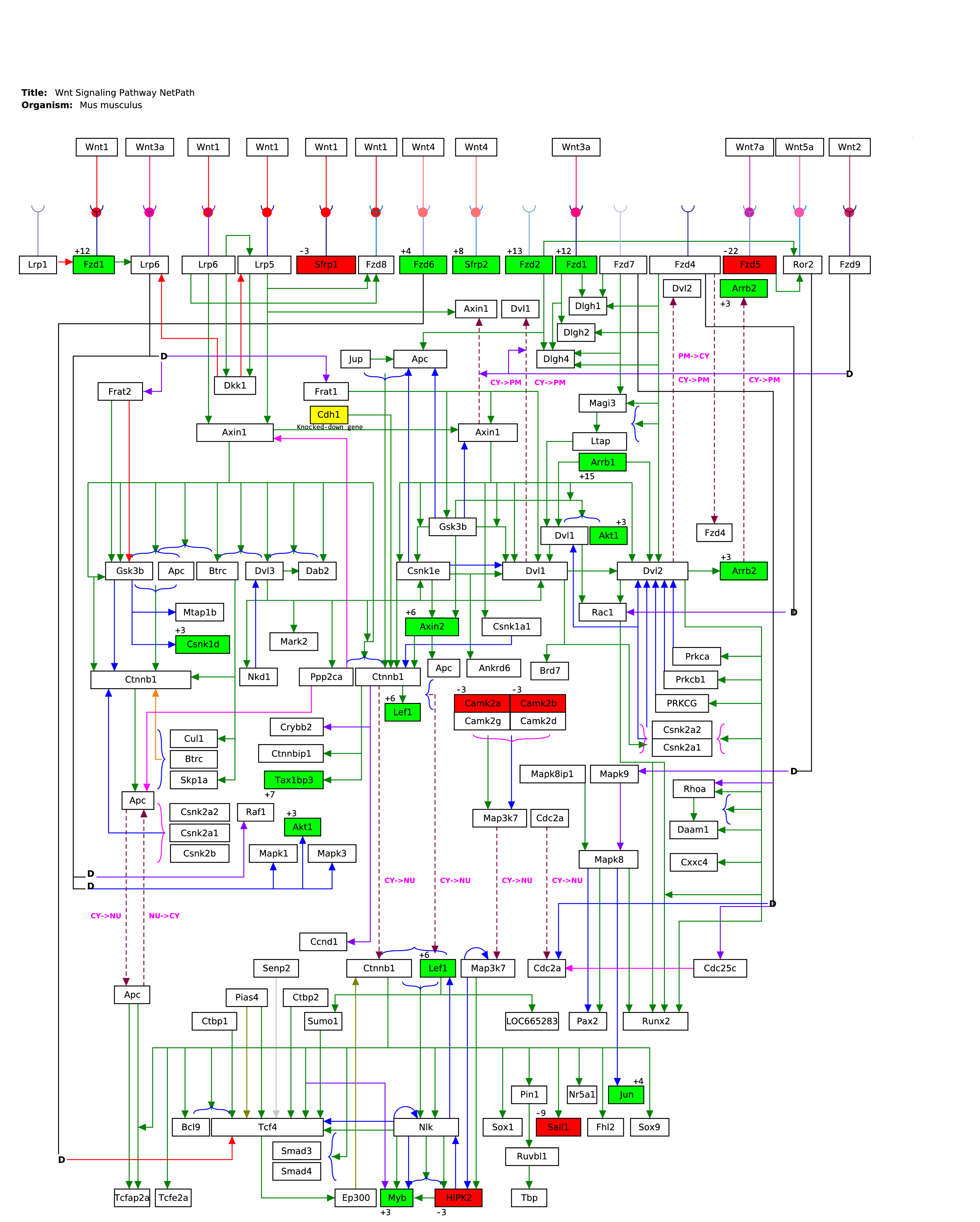

Supplement: Figure S4 — Network of transcripts associated with the Wnt signalling pathway. Transcripts exhibiting altered expression in Ecad-/- ES cells are highlighted: upregulated transcripts are shown in green and downregulated transcripts shown in red (with fold-change indicated by the number). Reproduced with kind permission from WikiPathways (http://creativecommons.org/licenses/by/3.0/). (TIF) [file pone.0021463.s004.tif]

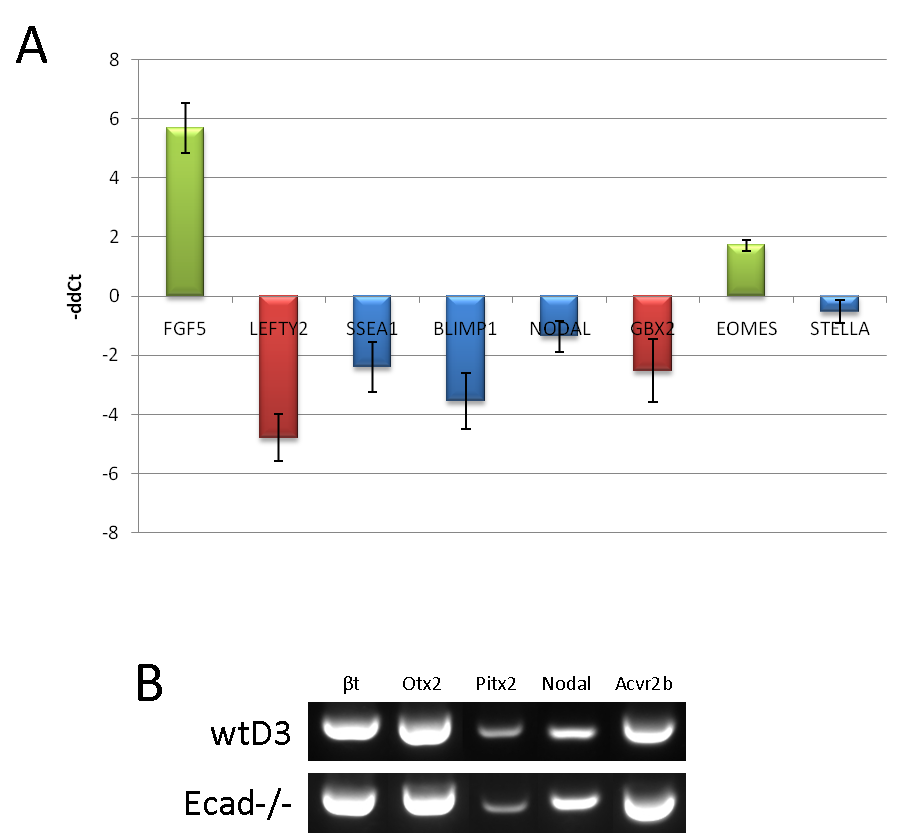

Supplement: Figure S5 — qPCR snd RT-PCR analysis of EpiSC-associated transcripts in Ecad-/- ES cells compared to wtD3 ES cells. qPCR analysis of FGF5, Eomes, Gbx2, Blimp1, Lefty2, Stella/Dppa3, SSEA-1 and Nodal in Ecad-/- ES cells compared to wtD3 ES cells (A). This analysis confirms the results of the microarray data. B. RT-PCR analysis of Otx2, Pitx2, Nodal and Acvr2b demonstrating similar expression of these transcripts in Ecad-/- and wtD3 ES cells. (βt = β-tubulin, loading control). (TIF) [file pone.0021463.s005.tif]
