# Supplementary material for: E-Cadherin Acts as a Regulator of Transcripts Associated with a Wide Range of Cellular Processes in Mouse Embryonic Stem Cells
Source: PLoS One. 2011 Jul 14;6(7):e21463. doi: 10.1371/journal.pone.0021463 (PMC3136471; doi:10.1371/journal.pone.0021463)
Supplement: Table S1 — Primer sequences for RT-PCR analysis. (DOC) [file pone.0021463.s006.doc]

| **Name** | **Forward Primer** | **Reverse Primer** |
| --- | --- | --- |
| **Acvr2b** | CATCATCACGTGGAACGAAC | CTTGTGGACAACCACCTCCT |
| **β-tubulin** | GGAACATAGCCGTAAACTGC | TCACTGTGCCTGAACTTACC |
| **Brachyury (T)** | CATTACACACCACTGACG | GATATAGGACCCTACCTAGC |
| **CRTR-1** | GGGCCCGCTTGCCGCCCCTA | CCCAGGCCGACTCCACCGCCA |
| **Dax1 (Nr0b1)** | TCCGGCGGGCAAGCCAGGTCC | GGCCAGCCGGCAGCCCCTCT |
| **Dazl** | GGCGACCCTCAGCACGCTCGCTT | TGCTGGCCTCCCTGGAGACAGCTGAA |
| **Fbxo15** | GGCCTCCTGGTGGGGCTGTGGC | GCCGGCTGCGGGAAATCAGGAAGGC |
| **FGF5** | GGCAGAAGTAGCGCGACGTT | TCCGGTTGCTCGGACTGCTT |
| **Klf4** | TGCGCCACCTCCCACGGCCC | TGGGGAGCTGCCGCCCCAGG |
| **Myc** | TGCGACGAGGAAGAGAATTT | AACCGCTCCACATACAGTCC |
| **Nodal** | ACGTTCACCGTCATTCCTTC | TCAGCTTCCCAAAGCAAAGT |
| **Otx2** | GGCTATGCTGGCTCAACTTC | GTCCATTTCAGGTTGCTGGT |
| **Pitx2** | CTGGAAGCCACTTTCCAGAG | CACCATGCTGGACGACATAC |
| **Rex-1** | AGGGCCGGGCGGAAGAGCCC | AGCTCGCCCCAACCCTCAGCGG |
| **Tdgf-1** | CGGAGATCTTGGCTGCTAAC | GGTCCAAATTCAAACGCACT |
