# Supplementary material for: E-Cadherin Acts as a Regulator of Transcripts Associated with a Wide Range of Cellular Processes in Mouse Embryonic Stem Cells
Source: PLoS One. 2011 Jul 14;6(7):e21463. doi: 10.1371/journal.pone.0021463 (PMC3136471; doi:10.1371/journal.pone.0021463)
Supplement: Table S2 — Primer sequences for qPCR analysis. (DOC) [file pone.0021463.s007.doc]

| **Name** | **Forward Primer** | **Reverse Primer** |
| --- | --- | --- |
| **Blimp1** | ccacagtgccttctccctta | gggggactactctcgtcctt |
| **Eomes** | cctggtggtgttttgttgtg | tttaatagcaccgggcactc |
| **FGF5** | aaaacctggtgcaccctaga | catcacattcccgaattaagc |
| **Gbx2** | cacggggacttttcgtctc | ggaacgctgcgctcatag |
| **Lefty-2** | gggagggagacatctcagaa | ttaggagaaatgggaacaacg |
| **Mrp L19** | acccctatgccagtggaaa | tccctgatcgcttgatgc |
| **Nodal** | caagttccaggtggacttcaa | ctgcttggggtagatgatcc |
| **Stella/Dppa3** | cttgttccgagctagcttttg | tcatccaaagcgtcctcttc |
