# Supplementary material for: E-Cadherin Acts as a Regulator of Transcripts Associated with a Wide Range of Cellular Processes in Mouse Embryonic Stem Cells
Source: PLoS One. 2011 Jul 14;6(7):e21463. doi: 10.1371/journal.pone.0021463 (PMC3136471; doi:10.1371/journal.pone.0021463)
Supplement: Table S3 — Signalling pathways identified in the microarray analysis as exhibiting significant alterations in Ecad-/- compared to wtD3 ES cells. (DOC) [file pone.0021463.s008.doc]

| **Category** | **Term** | **Count** | **%** | **PValue** | **FE** |
| --- | --- | --- | --- | --- | --- |
| mmu05200 | Pathways in cancer | 72 | 2.46 | 1.28E-05 | 1.6 |
| mmu04010 | MAPK signaling pathway | 59 | 2.02 | 9.32E-05 | 1.6 |
| mmu04510 | Focal adhesion | 45 | 1.54 | 4.61E-04 | 1.7 |
| mmu04310 | Wnt signaling pathway | 31 | 1.06 | 1.51E-02 | 1.5 |
| mmu04530 | Tight junction | 29 | 0.99 | 1.26E-02 | 1.6 |
| mmu04722 | Neurotrophin signaling pathway | 26 | 0.89 | 4.15E-02 | 1.5 |
| mmu04912 | GnRH signaling pathway | 25 | 0.85 | 2.00E-03 | 1.9 |
| mmu04916 | Melanogenesis | 23 | 0.79 | 1.30E-02 | 1.7 |
| mmu04540 | Gap junction | 23 | 0.79 | 1.92E-03 | 2.0 |
| mmu04512 | ECM-receptor interaction | 19 | 0.65 | 2.67E-02 | 1.7 |
| mmu04115 | p53 signaling pathway | 19 | 0.65 | 3.83E-03 | 2.0 |
| mmu04640 | Hedgehog signaling pathway | 18 | 0.62 | 5.09E-04 | 2.5 |
| mmu00480 | Glutathione metabolism | 18 | 0.62 | 3.08E-04 | 2.6 |
| mmu04920 | Adipocytokine signaling pathway | 17 | 0.58 | 1.50E-02 | 1.9 |
| mmu00520 | Amino sugar and nucleotide sugar metabolism | 16 | 0.55 | 4.15E-04 | 2.7 |
| mmu00565 | Ether lipid metabolism | 12 | 0.41 | 4.95E-03 | 2.5 |
| mmu00071 | Fatty acid metabolism | 11 | 0.38 | 7.45E-02 | 1.8 |
| mmu00310 | Lysine degradation | 10 | 0.34 | 9.36E-02 | 1.8 |
| mmu00051 | Fructose and mannose metabolism | 10 | 0.34 | 5.41E-02 | 2.0 |
