# Supplementary material for: E-Cadherin Acts as a Regulator of Transcripts Associated with a Wide Range of Cellular Processes in Mouse Embryonic Stem Cells
Source: PLoS One. 2011 Jul 14;6(7):e21463. doi: 10.1371/journal.pone.0021463 (PMC3136471; doi:10.1371/journal.pone.0021463)
Supplement: Table S4 — 20 most upregulated probes in wtD3 vs Ecad-/- compared to wtES vs EpiSCs. (FC = fold-change) (DOC) [file pone.0021463.s009.doc]

| **Gene** | **FC (wtD3 vs Ecad-/-)** | **q value** | **FC (wtES vs EpiSCs)** | | **q value** |
| --- | --- | --- | --- | --- | --- |
| **Serpina3m** | 164.6588 | 8.07E-07 | -6.05666 | NS | |
| **Btla** | 64.43393 | 9.36E-05 | -1.02855 | NS | |
| **Slc39a8** | 55.47226 | 1.86E-05 | 75.3407 | 0.005734 | |
| **Fgf5** | 51.91807 | 7.47E-06 | 231.585 | 0.021443 | |
| **Psors1c2** | 49.99709 | 3.66E-05 | -1.30604 | NS | |
| **BC064078** | 46.05629 | 1.44E-05 | 13.9072 | 0.006106 | |
| **Epha1** | 40.36505 | 1.82E-05 | 4.21463 | NS | |
| **Fst** | 37.72712 | 1.38E-05 | 6.51082 | 0.049243 | |
| **Chst1** | 34.44918 | 0.000554 | 23.9381 | 0.02694 | |
| **Galr2** | 31.45696 | 7.84E-06 | 3.32926 | NS | |
| **Gpr37** | 28.8434 | 7.36E-05 | 18.8153 | 0.00949 | |
| **Cd44** | 28.13673 | 4.07E-05 | 3.31627 | NS | |
| **Cd59a** | 26.54754 | 7.12E-06 | 24.7677 | 0.007797 | |
| **Palld** | 26.4357 | 0.000283 | 1.01995 | NS | |
| **Ms4a4d** | 26.42112 | 1.19E-05 | 1.88149 | NS | |
| **Slc5a5** | 25.55652 | 4.40E-05 | 2.09249 | NS | |
| **Calcr** | 25.47899 | 0.000214 | 5.40456 | 0.011205 | |
| **St8sia4** | 25.15873 | 0.001437 | 1.51364 | 0.038939 | |
| **Slc16a3** | 24.9354 | 0.000861 | 1.88766 | NS | |
| **Nes** | 24.3004 | 0.003982 | 3.53297 | NS | |
