# Supplementary material for: E-Cadherin Acts as a Regulator of Transcripts Associated with a Wide Range of Cellular Processes in Mouse Embryonic Stem Cells
Source: PLoS One. 2011 Jul 14;6(7):e21463. doi: 10.1371/journal.pone.0021463 (PMC3136471; doi:10.1371/journal.pone.0021463)
Supplement: Table S5 — 20 most downregulated probes in wtD3 vs Ecad-/- compared to wtES vs EpiSCs. (DOC) [file pone.0021463.s010.doc]

| **Gene** | **FC (wtD3 vs Ecad-/-)** | **q value** | **FC (wtES vs EpiSCs)** | **q value** |
| --- | --- | --- | --- | --- |
| **Esrrb** | -578.905 | 6.52E-07 | -82.5271 | 0.010589 |
| **Laptm5** | -526.866 | 3.79E-07 | -32.4319 | 0.015387 |
| **Nr0b1** | -450.485 | 9.77E-06 | -42.8288 | NS |
| **Pycard** | -448.514 | 8.36E-08 | -146.354 | 0.005854 |
| **Lgals3** | -444.904 | 7.22E-07 | -2.87589 | 0.049857 |
| **Calml4** | -401.914 | 9.20E-07 | -40.5604 | 0.020279 |
| **Zfp42** | -353.01 | 1.10E-06 | -1282.4 | 0.005832 |
| **Rnf17** | -286.466 | 1.53E-06 | -64.259 | 0.002822 |
| **Mras** | -280.27 | 1.46E-05 | -6.18893 | NS |
| **Lrrc34** | -260.24 | 9.90E-06 | -15.2251 | 0.020479 |
| **Slc38a4** | -259.482 | 3.07E-07 | -3.74646 | 0.017359 |
| **Cdyl2** | -251.768 | 3.07E-07 | -28.6657 | 0.00949 |
| **Jam2** | -235.072 | 3.07E-07 | -16.7514 | 0.012552 |
| **Hsf2bp** | -230.136 | 1.30E-07 | -3.29443 | 0.028772 |
| **Morc1** | -217.858 | 1.94E-05 | -542.091 | 0.005829 |
| **Aard** | -205.228 | 1.57E-05 | -2.05783 | NS |
| **2410004A20Rik** | -184.328 | 6.16E-05 | -180.663 | 0.006353 |
| **Epas1** | -173.216 | 1.97E-06 | -10.7295 | 0.011197 |
| **Tcl1** | -164.062 | 3.79E-07 | -12.1229 | 0.023622 |
| **2210016F16Rik** | -159.627 | 3.19E-07 | -5.35047 | NS |
