# Supplementary material for: E-Cadherin Acts as a Regulator of Transcripts Associated with a Wide Range of Cellular Processes in Mouse Embryonic Stem Cells
Source: PLoS One. 2011 Jul 14;6(7):e21463. doi: 10.1371/journal.pone.0021463 (PMC3136471; doi:10.1371/journal.pone.0021463)
Supplement: Table S6 — 20 most upregulated probes in wtES vs EpiSCs compared to wtD3 vs Ecad-/- ES cells. (FC = fold-change) (DOC) [file pone.0021463.s011.doc]

| **Gene** | **FC (wtES vs EpiSCs)** | **q value** | **FC (wtD3 vs Ecad-/-)** | **q value** |
| --- | --- | --- | --- | --- |
| **Cer1** | 5414.42 | 0.008002 | -1.00923 | NS |
| **Lhx1** | 462.157 | 0.011118 | -1.00305 | NS |
| **Asb4** | 318.518 | 0.005832 | -1.06776 | NS |
| **1500016L03Rik** | 287.765 | 0.011828 | -1.01103 | NS |
| **Cyp26a1** | 279.324 | 0.011 | 4.307831 | 0.001098 |
| **Rgs8** | 264.88 | 0.016344 | -1.00723 | NS |
| **Fgf5** | 231.585 | 0.021443 | 51.91807 | 7.47E-06 |
| **Foxa2** | 178.065 | 0.007191 | -1.00691 | NS |
| **Vtn** | 137.367 | 0.017767 | -1.00701 | NS |
| **Dgkk** | 127.876 | 0.018458 | -1.22274 | NS |
| **Sox17** | 117.287 | 0.014226 | -1.62967 | NS |
| **Dkk1** | 110.846 | 0.010659 | 1.08563 | NS |
| **Gsc** | 101.256 | 0.01653 | -1.02284 | NS |
| **Amot** | 94.7964 | 0.016054 | 4.784495 | 0.000139 |
| **Slc39a8** | 75.3407 | 0.005734 | 55.47226 | 1.86E-05 |
| **F5** | 74.1338 | 0.021118 | 1.114759 | NS |
| **Pcdh8** | 72.5662 | 0.023979 | 1.022659 | NS |
| **Frzb** | 70.0272 | 0.023335 | 1.23571 | NS |
| **Flt1** | 65.1831 | 0.014085 | 1.73046 | NS |
| **Slc35d3** | 57.3786 | 0.005829 | -1.0106 | NS |
