# Supplementary material for: E-Cadherin Acts as a Regulator of Transcripts Associated with a Wide Range of Cellular Processes in Mouse Embryonic Stem Cells
Source: PLoS One. 2011 Jul 14;6(7):e21463. doi: 10.1371/journal.pone.0021463 (PMC3136471; doi:10.1371/journal.pone.0021463)
Supplement: Table S7 — 20 most downregulated probes in wtES vs EpiSCs compared to wtD3 vs Ecad-/- ES cells. (FC = fold-change) (DOC) [file pone.0021463.s012.doc]

Table S7

| **Gene** | **FC (wtD3 vs Ecad-/-)** | **q value** | **FC (wtES vs EpiSCs)** | **q value** |
| --- | --- | --- | --- | --- |
| **Zfp42** | -1282.4 | 0.005832 | -353.01 | 1.10E-06 |
| **Stra8** | -1125.37 | 0.002189 | -26.0076 | 0.005594 |
| **Pramef12** | -1100.18 | 1.89E-04 | -16.4224 | 0.004661 |
| **Mael** | -682.744 | 0.006686 | -74.6738 | 5.19E-05 |
| **Syce1** | -678.394 | 0.005194 | -131.769 | 8.36E-08 |
| **Dppa4** | -617.929 | 0.006068 | -38.9074 | 3.60E-05 |
| **Fmr1nb** | -604.298 | 7.69E-04 | -6.35426 | 0.00811 |
| **Psma8** | -557.559 | 0.008723 | -50.9694 | 4.91E-06 |
| **Morc1** | -542.091 | 0.005829 | -217.858 | 1.94E-05 |
| **Fkbp6** | -443.864 | 0.005993 | -6.26377 | 0.006346 |
| **1700061G19Rik** | -413.943 | 0.007568 | -4.86664 | 0.040545 |
| **Sycp3** | -386.742 | 0.004625 | -17.0715 | 6.16E-05 |
| **D1Pas1** | -314.21 | 0.00214 | -54.7894 | 4.34E-05 |
| **Dppa3** | -311.331 | 0.018088 | -6.11967 | 0.001129 |
| **Ddx4** | -256.439 | 0.002822 | -105.139 | 4.82E-07 |
| **Dazl** | -253.748 | 0.009324 | -24.4682 | 0.000693 |
| **Tex14** | -240.807 | 0.001987 | -62.4421 | 2.02E-06 |
| **Tdh** | -238.29 | 0.005661 | -21.7521 | 2.79E-06 |
| **Six6os1** | -207.395 | 0.001907 | -5.74258 | 0.002635 |
| **2410004A20Rik** | -180.663 | 0.006353 | -184.328 | 6.16E-05 |
