# Supplementary material for: E-Cadherin Acts as a Regulator of Transcripts Associated with a Wide Range of Cellular Processes in Mouse Embryonic Stem Cells
Source: PLoS One. 2011 Jul 14;6(7):e21463. doi: 10.1371/journal.pone.0021463 (PMC3136471; doi:10.1371/journal.pone.0021463)
Supplement: Text S1 — (DOCX) [file pone.0021463.s013.docx]

**Supplementary Text**

**Materials and Methods**

*Proliferation assays*

wtD3 and E-cadherin-/- ES cells were grown in adherent culture in FCS+LIF and total cumulative viable cell numbers determined over 5 days using an automated cell counter (Innovatis) with trypan blue exclusion.

*Cell cycle analysis*

wtD3 and E-cadherin-/- ES cells were grown in adherent culture in FCS+LIF, trypsinised, washed twice in PBS and resuspended at 1 x 10^6^ cells/ml in FACS buffer (PBS + 0.1% BSA). Cells were fixed in cold ethanol (3ml ethanol/ml of cell suspension) and left for 1h at 4°C. Cell were washed twice with PBS and resuspended in 1ml of propidium iodide staining solution (Sigma, Dorset, UK. P 4170) containing 50μl of RNaseA stock solution and incubated at 4°C for 3h. Cell fluorescence was analysed using a Becton Dickinson FACScaliber. Viable cells were gated using forward and side scatter and the data represent cells from this population.

**Supplementary Discussion**

***Transcript alterations associated with specific gene ontology (GO) terms in Ecad-/- ES cells***

*Primary metabolic processes (BP GO: 0044238)*

In our analysis, 807 genes differentially expressed between Ecad-/- and wtD3 ES cells are associated with metabolic processes of cellular molecules, including amino acids, proteins, carbohydrates, lipids and nucleic acids. One of the genes up regulated in this group, Fgf-binding protein 1 (Fgfbp1), functions to protect Fgf-1 and -2 from acid inactivation [1] and can enhance their mitogenic effects. We have shown that Fgf-2 is required for optimal self-renewal of E-cadherin-/- ES cells and it is possible that secretion of Fgfbp1 from these cells may play a function in this process.

*Multicellular organismal development (BP GO:0007275)*

349 genes of this subgroup were represented in the Ecad-/- ES cell microarray and are associated with a variety of developmental processes such as organ morphogenesis (e.g. vasculogenesis, kidney, lungs, muscle) and hematopoiesis. There is a prevalence of genes associated with the development of both the central and peripheral nervous system. Within this group, Follistatin was significantly up-regulated in Ecad-/- ES cells compared to wtD3 ES cells (+38 FC). Follistatin is a regulator of Activin A and other members of the TGFβ superfamily [2]. We have previously demonstrated that Ecad-/- ES cells maintain pluripotency via Activin A and Nodal, suggesting that Follistatin may function to negatively regulate levels of Activin A response in these cells. Interestingly, the Activin beta-B subunit (Inhbb) was significantly down-regulated in Ecad-/- compared to wtD3 ES cells (-546 fold change), suggesting that this gene plays no role in maintaining Ecad-/- ES cell pluripotency.

*Cell differentiation (BP GO:0030154)*

Of interest in this subgroup is Angiopoietin-like 4 (Angptl4), which was down-regulated 70-fold in Ecad-/- ES cells compared to wtD3. Angptl4 inhibits lipoprotein lipase activity, thereby retarding lipoprotein catabolism. In addition, it has been shown that forced expression of Angptl4 in mouse melanoma cells prevented tumour cell extravasation from the circulation to the lungs and reduced migration, invasion, adhesion and cytoskeletal organisation of the cells in vitro [3].

*Cell adhesion (BP GO:0007155)*

Of the 107 genes represented in this group, some are involved in both cell-cell and cell-substrate adhesions, while others are cell adhesion regulators. Two genes are associated with integrin-mediated adhesion (CD24a antigen and intercellular adhesion molecule1, Icam1). Two immediate-early genes (IEGs), which are activated in cells prior to new protein synthesis following extracellular stimulation, were found to be up-regulated in this group. Cysteine-rich, Angiogenic Inducer 61 (Cyr61; +17-fold change) promotes endothelial cell adhesion via integrins and its expression is associated with increased tumorigenicity [4]. Connective tissue growth factor (Ctgf) is a mitogen and a key mediator of tissue fibrosis [5] that was increased 12-fold in Ecad-/- ES cells.

*Apoptosis (BP GO:0006915)*

This group comprises both positive and negative regulators of cell death. Microarray analysis revealed down-regulation of PYD and CARD domain-containing protein (Pycard) in Ecad-/- ES cells, which exhibits a proapototic effect in leukaemia cell lines by increasing their susceptibility to chemotherapeutic drugs [6]. The Pycard promoter has been found to be methylated in cancer cell lines and forced expression of Pycard in breast cancer cell lines resulted in inhibition of growth and colony survival [7]. Receptor-Interacting Serine/Threonine Linase 3 (RIPK3) exhibited 34-fold decreased expression in Ecad-/- ES cells. RIPK3 is a death domain adaptor protein that activates caspases via Tumour Necrosis Factor Receptor-1. An increased apoptosis-resistance, due to both downregulation of pro-apoptotic transcripts and up-regulation of transcripts associated with anti-apoptotic effects in Ecad-/- ES cells might be an interesting subject for future studies and correlate with the metastatic transformation of cancer cells, where loss of E-cadherin appears to be a fundamental process.

*Regulation of cell cycle (BP GP:0051726)*

In our analysis, we found genes involved in cell cycle checkpoints as well as regulators of various phases (e.g. S phase, meiotic and mitotic cycles). Genes represent both positive and negative regulators with a slight bias for the latter. Cyclic dependent kinase inhibitor 1c (Cdkn1c/p57(KIP2)) was down-regulated 127-fold in Ecad-/- ES cells. Cdkn1c is an inhibitor of G1 cyclin/Cdk complexes and a negative regulator of cellular proliferation [8].

*Transcription factor binding (MF GO:0008134)*

Several transcripts encoding chromatin remodelling proteins were down-regulated in Ecad-/- ES cells. SWI/SNF-Related Matrix-Associated Actin-Dependent Regulator of Chromatin (Smarc)-D3 exhibited 98-fold decreased expression. SmarcD3 functions to recruit chromatin remodelling complexes to specific enhancers during organogenesis, and is known to regulate cardiac and skeletal muscle differentiation [9]. Histone acetyl transferase MYST4 (-22 FC) is a ubiquitously expressed protein containing transcriptional repression and activation domains and may function as a regulator of both positive and negative transcription.

**Supplementary Bibliography**

1. Wu DQ, Kan MK, Sato GH, Okamoto T, Sato JD (1991) Characterization and molecular cloning of a putative binding protein for heparin-binding growth factors. J Biol Chem 266: 16778-16785.

2. Schneyer A, Sidis Y, Xia Y, Saito S, del Re E, et al. (2004) Differential actions of follistatin and follistatin-like 3. Mol Cell Endocrinol 225: 25-28.

3. Galaup A, Cazes A, Le Jan S, Philippe J, Connault E, et al. (2006) Angiopoietin-like 4 prevents metastasis through inhibition of vascular permeability and tumor cell motility and invasiveness. Proc Natl Acad Sci U S A 103: 18721-18726.

4. Babic AM, Kireeva ML, Kolesnikova TV, Lau LF (1998) CYR61, a product of a growth factor-inducible immediate early gene, promotes angiogenesis and tumor growth. Proc Natl Acad Sci U S A 95: 6355-6360.

5. Phanish MK, Winn SK, Dockrell ME Connective tissue growth factor-(CTGF, CCN2)--a marker, mediator and therapeutic target for renal fibrosis. Nephron Exp Nephrol 114: e83-92.

6. Masumoto J, Taniguchi S, Ayukawa K, Sarvotham H, Kishino T, et al. (1999) ASC, a novel 22-kDa protein, aggregates during apoptosis of human promyelocytic leukemia HL-60 cells. J Biol Chem 274: 33835-33838.

7. Conway KE, McConnell BB, Bowring CE, Donald CD, Warren ST, et al. (2000) TMS1, a novel proapoptotic caspase recruitment domain protein, is a target of methylation-induced gene silencing in human breast cancers. Cancer Res 60: 6236-6242.

8. Lee MH, Reynisdottir I, Massague J (1995) Cloning of p57KIP2, a cyclin-dependent kinase inhibitor with unique domain structure and tissue distribution. Genes Dev 9: 639-649.

9. Lickert H, Takeuchi JK, Von Both I, Walls JR, McAuliffe F, et al. (2004) Baf60c is essential for function of BAF chromatin remodelling complexes in heart development. Nature 432: 107-112.
